# Cognitive function in multiple sclerosis improves with telerehabilitation: Results from a randomized controlled trial

**DOI:** 10.1371/journal.pone.0177177

**Published:** 2017-05-11

**Authors:** Leigh E. Charvet, Jie Yang, Michael T. Shaw, Kathleen Sherman, Lamia Haider, Jianjin Xu, Lauren B. Krupp

**Affiliations:** 1 Department of Neurology, NYU School of Medicine, New York, New York, United States of America; 2 Department of Family, Population, and Preventive Medicine, Stony Brook Medicine, New York, New York, United States of America; 3 Taub Institute, Columbia University Medical Center, New York, New York, United States of America; 4 Department of Applied Mathematics and Statistics, Stony Brook Medicine, Stony Brook, New York, United States of America; University Medical Center Gottingen, GERMANY

## Abstract

Cognitive impairment affects more than half of all individuals living with multiple sclerosis (MS). We hypothesized that training at home with an adaptive online cognitive training program would have greater cognitive benefit than ordinary computer games in cognitively-impaired adults with MS. This was a double-blind, randomized, active-placebo-controlled trial. Participants with MS were recruited through Stony Brook Medicine and randomly assigned to either the adaptive cognitive remediation (ACR) program or active control of ordinary computer games for 60 hours over 12 weeks. Training was remotely-supervised and delivered through a study-provided laptop computer. A computer generated, blocked stratification table prepared by statistician provided the randomization schedule and condition was assigned by a study technician. The primary outcome, administered by study psychometrician, was measured by change in a neuropsychological composite measure from baseline to study end. An intent-to-treat analysis was employed and missing primary outcome values were imputed via Markov Chain Monte Carlo method. Participants in the ACR (n = 74) vs. active control (n = 61) training program had significantly greater improvement in the primary outcome of cognitive functioning (mean change in composite z score±SD: 0·25±0·45 vs. 0·09±0·37, p = 0·03, estimated difference = 0·16 with 95% CI: 0·02–0·30), despite greater training time in the active control condition (mean±SD:56·9 ± 34·6 vs. 37·7 ±23 ·8 hours played, p = 0·006). This study provides Class I evidence that adaptive, computer-based cognitive remediation accessed from home can improve cognitive functioning in MS. This telerehabilitation approach allowed for rapid recruitment and high compliance, and can be readily applied to other neurological conditions associated with cognitive dysfunction.

**Trial Registration:** Clinicaltrials.gov NCT02141386

## Introduction

Cognitive impairment occurs in up to 70% of all patients with multiple sclerosis (MS) affecting information processing, attention and learning [1, 2]. Despite longstanding recognition, it remains a troubling symptom without adequate treatment.

There has been limited study of cognitive rehabilitation in MS. Traditional approaches (e.g., compensatory strategies and drill-and-practice training) are costly and difficult to uniformly implement, but with some trials indicating benefit [3–6]. However, any cognitive training program requires multiple sessions administered across weeks or months. Due to the burdens of time and travel, the requirement for traveling to clinic for training often prevents access to treatment for many patients.

Recent computer-based cognitive training CT approaches use technological advances to deliver learning trials that are adapted to the individual user in real-time [7–9]. Rather than focusing on compensation, intensive repetitive exercise may actually improve cognitive ability at the processing level [8–10]. Further, participants may train on the computer from home[11], providing access for the many patients for whom repeated outpatient visits are not feasible. Adaptive cognitive remediation (ACR) programs have shown benefit in normal aging [12] and schizophrenia [13]. In MS, a large but underpowered controlled trial reported promising cognitive improvements [14]. Similarly, we found preliminary benefit in a small controlled pilot study to establish our protocol for this trial [11].

In this study, we tested the efficacy of a computer-based ACR program in MS against an active control comparison of ordinary computer games. Participants with MS and cognitive impairment were screened, enrolled, and randomized to either the ACR or active control training conditions. Training was completed from home for 12 weeks using a telerehabilitation protocol based on remote monitoring and supervision.

## Materials and methods

### Study design

This study was a double-blind, randomized, active-placebo-controlled trial. Institutional review board (IRB) approval was provided through Stony Brook University Hospital in Stony Brook, New York. Initial IRB approval was obtained April 11, 2013. Recruitment began September 10, 2013 through June 5, 2015 with last data collection September 9, 2015. The authors confirm that all ongoing and related trials for this drug/intervention are registered at clinicaltrials.gov, number: NCT02141386. Due to an administration error, registration occurred after enrollment was initiated.

### Participants

Enrollment criteria were designed to be as inclusive as possible given that the computer-based CT programs may be available to a wide range of participants through prescription or commercial access. Participants included those meeting diagnostic criteria for MS [15] (any subtype) and scoring one or more standard deviations below published normative data on the Symbol Digit Modalities Test or SDMT [16]. The SDMT is considered a sensitive measure of cognitive involvement in MS with performance acting as an accurate predictor of generalized neuropsychological functioning [17].

To ensure adequate understanding of the CT instructions and valid administration of the neuropsychological testing (currently available in English language only), participants were required to have a reading recognition standard score of 85 or above (Wide Range Achievement Test Third Edition or WRAT-3) (24), and have learned English by age 12 years. Participants were also required to have adequate visual, auditory, and motor capacity to operate computer software. Additional inclusion criteria were no anticipated medication changes during the course of the three-month study period, and no relapses or steroids in the previous month.

Exclusion criteria were defined as history of any developmental disorders, conditions other than MS associated with cognitive impairment, a primary psychiatric disorder, any serious medical conditions, alcohol or substance use disorder, and also history of use of computer-based CT developed by Posit Science (the developer of our study program). Eligible participants provided written, informed consent prior to all study procedures.

### Randomization and masking

Both the participant and study psychometricians were blinded to treatment condition. As any training could be potentially beneficial, participants were told they would be randomly assigned to one of two training programs that were being compared. A study technician, separate from study psychometrician, followed the random allocation sequence to assign participants to a study condition and prepare study equipment and materials.

Eligible and consented participants were randomly assigned to the ACR or active control condition using stratified, permuted, block randomization generated by the study statistician (Dr. Yang). Strata were based on three levels for the factors of age (<35, 35 to 50, and >50 years), WRAT-3 (24) reading recognition standard score (as an estimate of premorbid intellectual functioning (25): <85, 85 to 115,>115) and SDMT age-normative z score (as an estimate of current cognitive impairment: ≤-3•00–2•00 to -2•99, and -1•00 to -1•99). A designated study technician enrolled and assigned participants to either condition and prepared all study laptops. The study technician that assigned a participant’s condition was not involved in the collection of data at baseline or study end visits. Study psychometricians collected the outcome data and were blinded to participant condition.

*The ACR condition* was an online adaptive cognitive training program developed by Posit Science Corporation [18]. The program was a research version of the BrainHQ program, and offered a portal dedicated to the study, central management of study participation and metrics, and a set of 15 exercises targeting speed, attention, working memory, and executive function through the visual and auditory domains.

Each exercise was adaptive, employing a Bayesian algorithm operating on a trial-by-trial basis to increase the challenge as participants performed correctly and to reduce challenge as participants performed incorrectly, consequently participants generally performed ~80% of trials correct. For example, a processing speed exercise adapts presentation time to slower or faster rates, while a working memory exercise adapts items in the working memory span higher or lower. This design allows for an initial low level of challenge, with adjustments applied on an individualized basis as learning and abilities improve over time. This feature maintains a high level of challenge without reaching a level of failure or frustration, and consistently engages the user in task performance.

Each exercise employed multiple stimulus sets designed to span relevant dimensions of real-world stimuli. For example, auditory exercises employed stimuli related to human speech perception that were initially slowed and later speeded, while visual exercises initially employed simple high contrast stimuli and later provided stimuli that were naturalistic and low contrast.

Over the course of daily trials, a participant is required to attend to stimuli, detect novel stimuli, and generally receive a reward (after a correct trial). These aspects of training were designed to repetitively engage cholinergic selective attention systems, noradrenergic novelty detection systems, and dopaminergic reward systems.

The goal of the training exercises is to improve the speed and accuracy of brain information processing while engaging neuromodulatory systems, and in doing so, allow the generalization of training to improvement cognitive performance in real-world situations. The exercises were sequenced across the duration of a participant's involvement such that auditory exercises were delivered first, with visual exercises coming later. Each daily training session consisted of four exercises chosen from an active set of six; when all of the content in an exercise was completed (typically over a number of days), that exercise was withdrawn from the schedule and the next exercise added to the active set of six.

*The active control condition* was a software gaming suite developed by Hoyle Puzzle and Board Games (2008 version) [19]. These games served as an active placebo control, designed to account for nonspecific treatment effects including interactions with research personnel, and computer-based game-playing. Previous trials have used similar games as an active control condition to demonstrate the specific effects of the targeted adaptive training program [20–22]. Participants were provided a set gaming schedule and were instructed to play games in an arrangement that mirrored to the active condition, with a schedule of four games per session for 15 minutes each following a set rotational sequence [11]. The games were selected for “face validity” as having cognitive benefit (e.g., word puzzles) but did not include the active condition’s program design features to drive learning or maintain user challenge.

### Procedures

Participants were instructed to train in their assigned condition for one hour per day, five days per week, over 12 weeks (targeting 60 hours of total program use). The training schedule for both conditions was predetermined, with both the ACR and active control condition having rotating sets of training games. Participants had ongoing access to technical support as well as a scheduled weekly check-in phone call. The unblinded study technician conducted these weekly check-in phone calls, as they were not involved in the administration of study outcome measures.

All participants used a study-provided 17” laptop computer, peripheral equipment including headphones, and a user guide with directions for the use of their assigned program, following procedures previously described [11]. All laptops were configured with a secure monitoring software program (“WorkTime” developed by NesterSoft, Inc) to monitor and record program compliance in real-time throughout the study. This software tracked and recorded all computer activity in real-time. Therefore, ongoing acquisition of data, such as the amount of time spent on games, informed the weekly phone contact.

Baseline and Study End Assessments: Neuropsychological measures were administered at baseline and repeated at study end. At the study end visit, the participants returned the study equipment. Participants were reimbursed $100 for completion of each of the two study assessment visits for a total of $200.

### Outcomes

Primary Outcome—Neuropsychological Composite Score: A battery of neuropsychological tests [23–28] was administered at baseline and study end visits (shown in Table 1) consisting of key tests that are commonly used to measure MS-related cognitive impairment. Alternate forms were used for each of the measures, with the order counterbalanced across participants. To provide a composite score of cognitive functioning, the main representative measure from each test (e.g., total learning across trials) was transformed to a z score based on published or manual-provided age-normative data. Only one key score per test was included (defined a priori) to avoid overrepresentation of any one test in the composite. The z scores for each test measure were averaged to result in a composite z score. Finally, for each participant, the difference between their baseline and study end composite z scores were calculated to serve as the primary outcome of change in cognitive performance.

**Table 1:** Primary outcome: Neuropsychological composite.

Neuropsychological Test	Cognitive Domain	Study Outcome
Paced Auditory Serial Addition Test (PASAT)^1^	Processing Speed	2 Second Trial
WAIS-IV Letter Number Sequence^2^	Working Memory	Total Score
WAIS-IV Digit Span Backwards^3^	Working Memory	Total Score
Selective Reminding Test (SRT^)^^4^	Verbal Learning	Total Recall across Learning Trials
Brief Visuospatial Memory Test-Revised (BVMT-R^)^^5^	Visual Learning	Total Recall across Learning Trials
Delis-Kaplan Executive Function System Trails	Visual Scanning	Composite (Average of Number and Letter Sequencing Trials)

^1^ Paced Auditory Serial Addition Test[23],

^2,3^ WAIS-IV Letter Number Sequencing and Digit Span[24],

^4^ Selective Reminding Test[25],

^5^ Brief Visuospatial Memory Test-Revised[26],

^6^ Delis-Kaplan Executive Function System Trails[27, 28].

To explore if any cognitive change appeared to be specific to any one measure, groups were also compared on change in the individual measures of the composite.

Program Compliance: As a secondary outcome, compliance was measured as a by two approaches: total time and number of compliant weeks. Compliance was defined as program use of 50% or more of target (i.e., 30 hours total) and secondarily as compliant weeks, or having at least 50% of the total study period (6 weeks or more) where there was at least 50% compliance for that week (2·5 hours or more) [11].

Self-Reported Change in Cognitive Functioning: As a secondary outcome measure, at study end, participants rated whether their cognition stayed the same (0), improved (1) or declined (-1) from baseline to study end.

### Statistical analysis

Sample size was estimated based on published trials of Posit training programs and from an initial pilot study of n = 10 MS participants. Data were entered using the Research Electronic Database Capture (REDCap) [29] system. A data monitoring committee did not oversee the study due to the lack of risks involved with the remediation. An intent-to-treat analysis was employed and all participants were included in analyses.

As pre-study planned, a linear mixed model adjusted for the three randomization stratification factors (age, WRAT-3 reading standard score, SDMT z-score) as covariates to decide whether the primary outcome score had changed significantly between the two study arms. The dependence structure of the two scores from each participant was modeled as compound symmetry.

There were five (3·74%) patients who had incomplete scores. To be conservative, sensitivity analysis was further performed to check the influence of these missing data. Two methods are used: one is stratified non-parametric test (van Elteren test) using complete cases only and other one is completed with multiple imputation procedure with the Markov Chain Monte Carlo (MCMC) method [30] applied to impute the missing values of the primary outcome (composite cognitive z-score). Variables used to impute the missing values of end point outcomes included all the participant’s demographic information (age, gender, race and ethnicity, year of education) and baseline test scores. These imputed data sets were then analyzed by linear mixed model and the results from these analyses were combined based on Rubin’s rule [31].

We hypothesized that two factors may predict treatment outcome for neuropsychological benefit: degree of estimated cognitive impairment at screening, as measured by the SDMT, and total time played in the assigned condition. Linear mixed models were applied to test if screening SDMT or total time played contributed to the change in the neuropsychological composite at study end visit. All analyses were performed in SAS 9·3 and significance level was set at p-value < 0·05.

## Results

**Fig 1:**
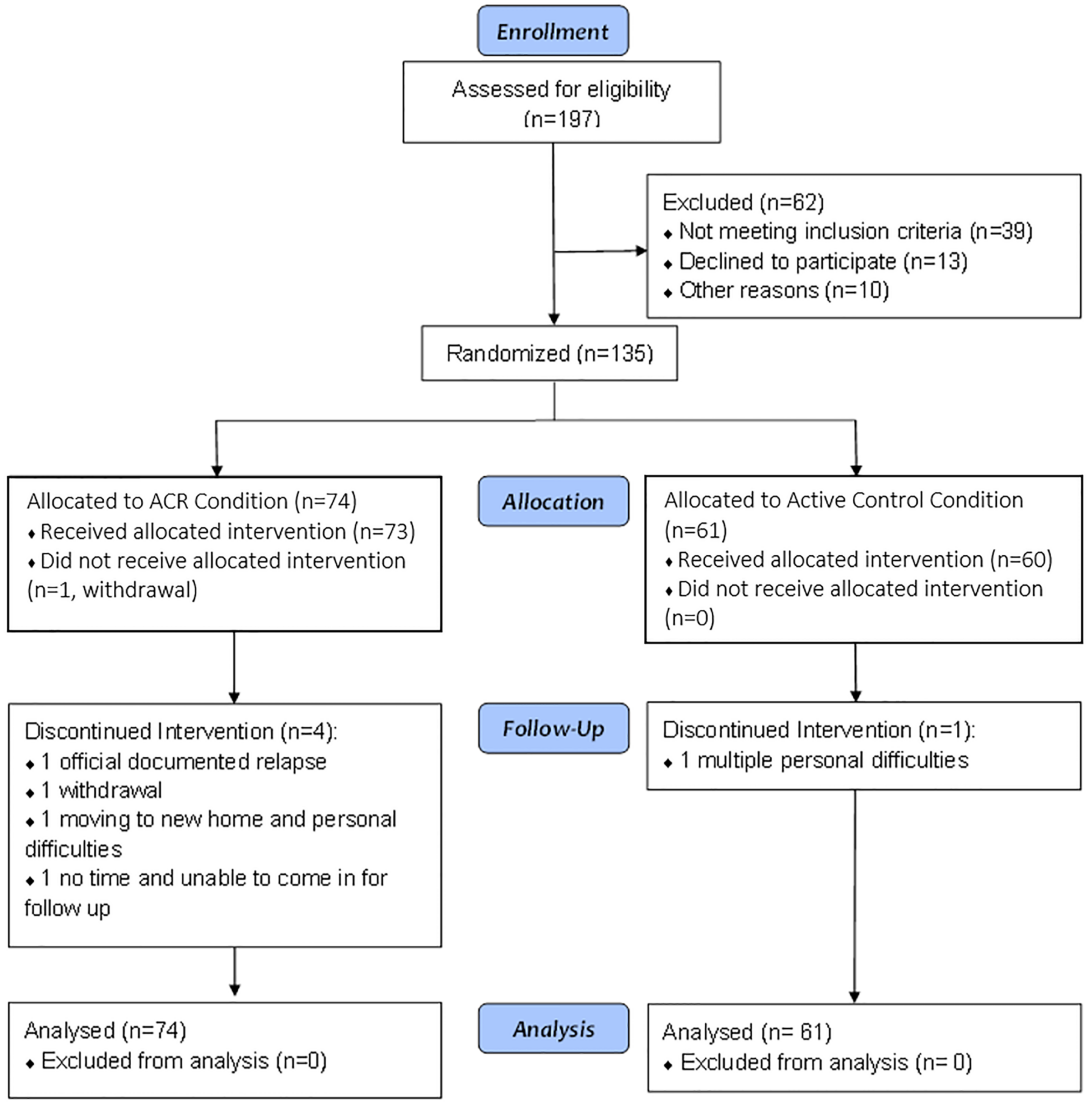
Consort flow diagram.

A total of n = 135 participants were enrolled between September 10, 2013 and June 5^th^, 2015 and all study visits were completed as of September, 9^th^, 2015. Fig 1 shows the enrollment and study flow, with n = 74 assigned to the ACR condition and n = 61 to the active control condition (with unequal samples due to the stratification requirements). Only five participants did not complete the study and all equipment was returned without damage. Reasons for withdrawal of the trial included acute relapse (n = 1) and medical and personal issues that prevented adherence to study procedures (n = 4).

Table 2 shows the demographic and clinical features and baseline neuropsychological performances of the participants in each condition. The participants were generally middle-aged (mean age±SD: 49·80 ± 12·45) and mostly women (77·04%). The majority (67·85%) of the group had relapsing remitting MS (RRMS), overall mild to moderate neurologic disability (median EDSS score of 3·5), and mean disease duration±SD of 12·62 ± 10·46 years. Based on screening SDMT scores, there was a mild-to-moderate degree of overall impairment (mean SDMT z-score±SD of -2·10 ± 0·99).

**Table 2:** Demographic and clinical characteristics.

Demographic Characteristics	ACR Condition (n = 74)	Active Control Condition (n = 61)	Full Sample (n = 135)
Sex (n,% Female)	50, 67·57%	54,88·52%	104,77·04%
Age (mean years±SD)	48±13	52±11·	50±12
Education (mean Years±SD)	14·82±2·37	15·05±2·55	14·93±2·45
Race (n, %):	-	-	-
White	63,85·14%	51,83·61%	114,84·44%
Black/African American	6,8·11%	4,6·56%	10,7·41%
Other/Unknown	5,6·76%	6,9·84%	11,8·15%
Ethnicity—Hispanic or Latino (n, %):	7, 9·86%	3,5·26%	10,7·81%
Clinical Characteristics	-	-	-
MS Subtype (n, %)			
Relapsing-Remitting	51 (69%)	39 (64%)	89 (66%)
Primary Progressive	3(<1%)	4 (<1%)	7 (<1%)
Secondary Progressive	20 (27%)	15 (23%)	35 (26%)
Disease Duration (mean years±SD)	11·9±10·9	13·5±10·0	12·62±10·46
EDSS (median score±IQR)	3·50±4·00	3·50±4·00	3·50±4·00
25 Foot Timed Walk (median seconds, min-max)	5·7 (3·5–28·75)	6·3 (3·25–24·75)	5·9 (3·25–28·75)
Screening SDMT z score (mean score±SD)	-2·10±0·99	-2·10±1·01	-2·10±0·99

As also indicated in Table 2, the stratification was successful and the groups were overall well-matched across all variables at baseline, only significantly differing with more men assigned to the ACR versus active control condition (24 of a total of 31 enrolled men).

The two groups did not differ in performance on any measures at baseline, with generally mild to moderate deficits on measures of information processing and memory (Composite score: ACR n = 74 vs. active control n = 61, mean±SD:-0·86±0·77 vs. -0·77±0·73, respectively, p = 0·4569). Change scores varied across both conditions, with a greater range in the active arm: ACR n = 70 vs active control n = 60, mean±SD:-0·25±0·45 vs. 0·09±0·37, Cohen’s *d* = 0·3883) as shown in Table 3. Under intent-to-treat analysis the active condition had a significantly higher change in the neuropsychological composite from baseline to study end using linear mixed model (estimated difference = 0·16 with 95% CI: 0·02–0·30, p = 0·0286). Such statistical significance was also found by using stratified nonparametric test (p = 0·0073) and multiple imputation (estimated difference = 0·16 with 95% CI: 0.02–0.30, p = 0·0299).

**Table 3:** Composite score differences between conditions.

Treatment Condition*	Mean ± SD baseline z-score	Mean ± SD end z-score	Mean ± SD change z-score
ACR	-0.77 ± 0.73	-0.68 ± 0.76	0·25 ± 0·45
Active Control	-0.86 ± 0.77	-0.60 ± 0.85	0·09 ± 0·37

* 5 subjects who dropped out the study did not have end z-scores and hence were not included in calculating end z-score and change in z-score.

**Fig 2:**
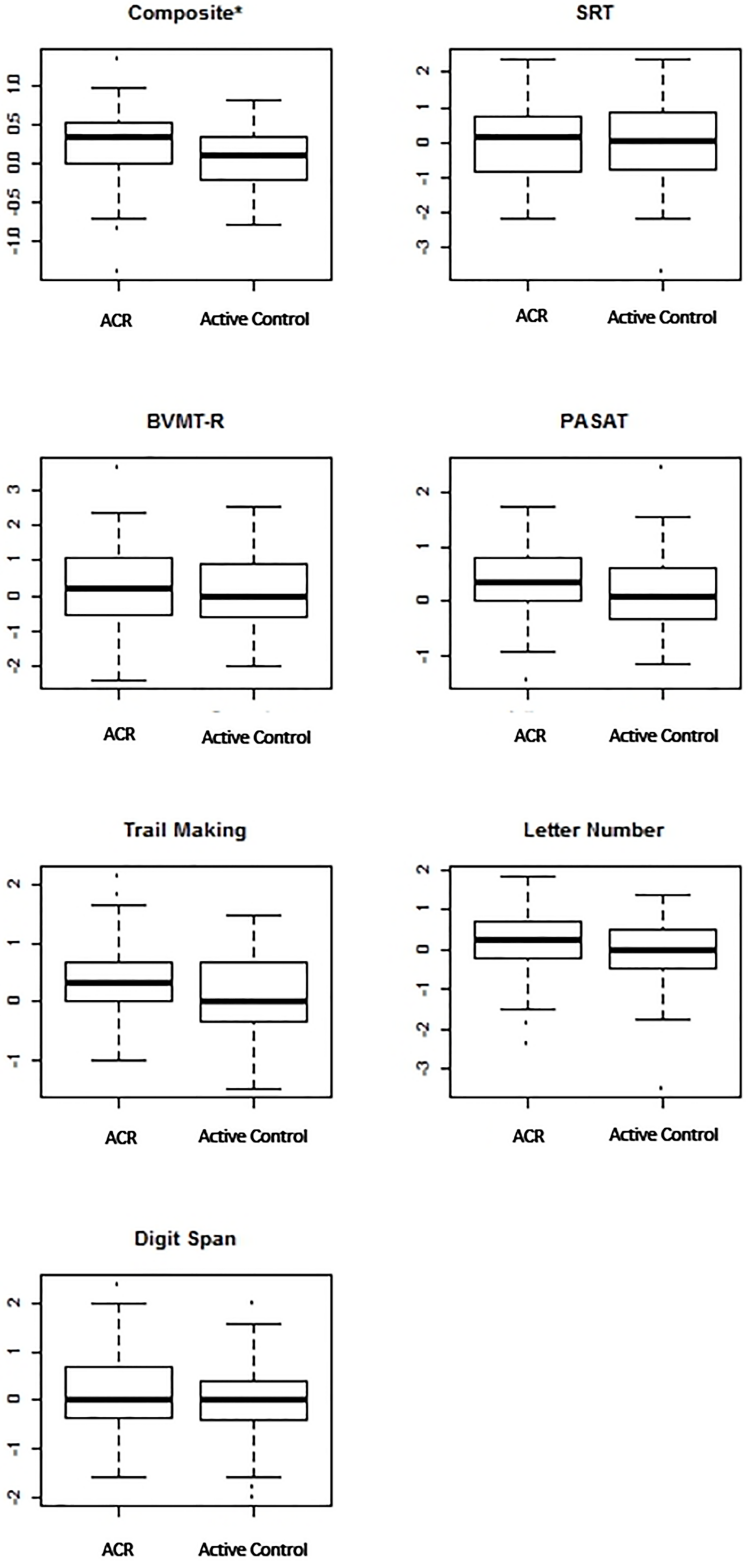
Change in z score across measures by condition. *Indicates a significant difference corresponding to a p value<0.05. Higher scores indicate improvement.

Among the individual measures, very few were found to have statistically significant improvement between the two groups (Fig 2 displays the change in performance on each measure in both groups). The active group was found to have significant improvement on the 2 second PASAT via the van Elteren test and a significant improvement was also found on the DKEFS as accounted by the linear mixed model, neither measure was found to be significantly improved by both models, unlike the larger composite improvement. Without the concordance of both models, we discarded these findings.

**Fig 3:**
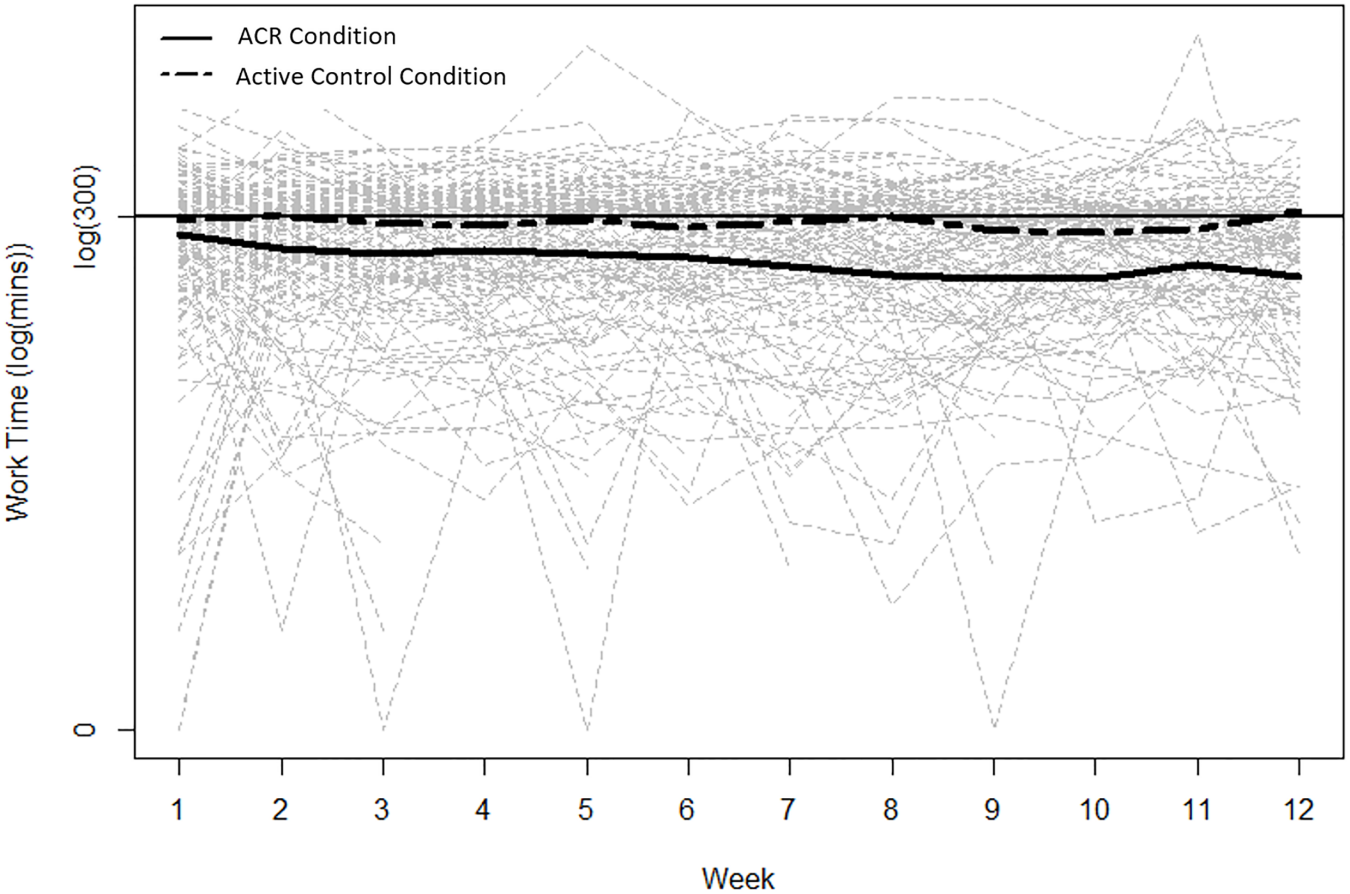
Total time spent in program by condition. *Greater time was spent in program by the active control condition (p = 0.006).

Compliance was high for the full sample, with n = 91 (67·4%) and n = 92 (68·15%) participants playing at least 50% of the goal depending on how compliance was defined (at least 6 compliant weeks or meeting or exceeding 30 hours of training time). However, versus those in the ACR condition, the active control condition group had greater compliance (active control n = 48 vs. ACR n = 43, 78·69% vs. 58·11% or active control n = 48 vs ACR n = 44, 78 ·69% vs. 59·46%) and spent significantly greater time in program than the active condition (Fig 3: ACR vs. active control, mean±SD = 37·74±23 ·78 vs. 56·95±34·53, p = 0·0056).

Neither total program time nor screening SDMT contributed to the change at study end performance. However, when considering different conditions separately there was a positive marginal correlation between the composite z-score change and total program time for the active condition (r = 0·25, p = 0·03) indicating a modest link between time spent playing the active program and magnitude of improvement on the neuropsychological composite score.

At study end, more active condition participants (56·7% vs. 31·0%) reported experiencing an improvement in cognition over the 12-week duration of the study (indicating a rating of 1·0, versus no change of 0·0, and -1·0 for decline: ACR vs. active control, mean±SD = 0·52±0·59 vs. 0·28±0·52, p = 0·007).

## Discussion

We found that 12 weeks of training with an ACR training program was superior to an active control of playing ordinary computer games for improving cognitive functioning in participants with MS. The benefit was measured by a change in a composite of neuropsychological tests and was modest overall. No one measure indicated a specific response to the training; instead, the majority of cognitive measures changed in a direction that favored the active program. This lack of specificity may be attributable to the diffuse effects of improved cognitive processing speed across the range of measures, mediated by individual differences in baseline performances.

The significantly greater benefit for the adaptive training program was found despite significantly less program training time. Participants in the active control condition trained an average of 19 hours more than those in the ACR program. Notably, in the active program only, time played was significantly associated with cognitive improvement.

Our findings are consistent with our prior pilot study (using a different adaptive training program for the same time period) [11] as well as a previous trial of a version of the study program in a smaller sample [14]. Additionally, our findings also concur with those reported in a recent meta-analysis that found modest cognitive benefit for healthy aging adults who underwent cognitive remediation, typically seeing the greatest benefit for cognitive domains that were trained most often [7]. The findings in this study are encouraging in that this is the largest clinical trial of cognitive remediation in an MS sample published as of yet and the results support the hypothesis that cognitive impairment in MS may be remediated [6, 11, 14]. Further, as baseline cognitive functioning as measured by the SDMT was not predictive of change in cognitive functioning overall or in response to the intervention, taken together the findings suggests that this intervention may be appropriate for MS participants with a wide range of cognitive problems.

With the advent of modern technological advances in healthcare, approaches towards rehabilitation, treatment, and cognitive remediation are transitioning to an online platform that can be adaptive and personalized. However, computerized cognitive training programs have not yet been evaluated thoroughly, with methodological criticisms raised for much of the prior work in this field [32]. Importantly, the current study overcomes the limitations of many previous studies (including small sample sizes, passive controls, and unrepresentative outcomes) and far exceeds the scope of other remote cognitive training studies in the field of MS.

A major advantage of this approach was providing access to the intervention from home. Our enabling of participants to access treatment from home allowed rapid study enrollment (n = 135 over 12 months), strong program compliance, and relatively low cost when considering for real-world use. For compliance and structured use, we believe that the remote supervision is a critical element. As was also seen in our pilot study [11], our relatively low rate of noncompliance and study withdrawal indicates the success of our remote approach. The remote supervision approach provides the patient with readily available assistance and reinforcement to keep a patient on target, especially in older, aging samples. Additionally, it is notable that all study equipment was returned, preserving overall treatment cost. The remote approach is especially exciting for the potential of opening up participation for studies of this kind for individuals who are largely home-based or who or struggling to maintain employment and reluctant to participate in rehabilitative activities that would interfere with their work and family time commitments.

Methodological challenges to the study included the broad parameters of the active study program and our dependence on the developers for the provision of the study program. Over the course of the study, there were centralized technical difficulties and platform changes and updates that may have affected our study users’ experience to varying degrees. While we included an active control comparison condition to control for computer use and game playing activity, we did not control for the higher-level features of the active program such as adaptive versus non-adaptive features and other design aspects to drive learning. At the end of study, the gender distribution was not balanced between the two study arms, which occurred randomly. However, there is no evidence to support a gender difference in any possible treatment effect and hence this imbalance will not affect our interpretation of the results.

It is not clear which aspects of the active program were therapeutic and how this program would compare to other available programs with similar design features. Future studies should be able to determine the precise nature of training and domain-specific improvement, baseline characteristics to predict response, and further explore titration and treatment time. Another unknown consideration is the duration of benefit. Once established, the cognitive benefit may require continued training to be sustained over time, and there may be a regression in functioning once the training is discontinued.

By purposefully including broad entry criteria we were able to study the home use of cognitive training programs in a real-world setting. The study was designed to approximate an application for individuals with MS interested in participating in a cognitive training program, initiated either through prescription or self-referral. Going forward, more careful study of patients with specific disease features will allow for targeted program adjustments.

An additional limitation is that measures of depression and fatigue were not included. Both symptoms are common in MS and known to influence cognitive functioning. Therefore, it would be important to both characterize the sample at baseline on these symptom features, as well as to measure change in the symptom severity following treatment and in relation to the presence or absence of cognitive benefit.

This study capitalizes on recent technological advances and provides an alternative route for cognitive remediation, through remote-supervision at home. This study supports the feasibility of computer-based cognitive remediation accessed from home, and demonstrates Class 1 efficacy of the treatment. Further trials may seek to determine which members of the MS population are most responding to benefit or, alternatively, how benefit can be enhanced or sustained through techniques such as medication, neuromodulation, or even exercise. The remote delivery and findings of cognitive benefit may be generalizable to other neurological conditions in which cognitive function is compromised and this study can serve as a model for these trials.

## Supporting information

S1 Checklist Consort 2010 checklist for clinical trials. (DOC)

S1 Dataset Minimal dataset. (CSV)

S1 Protocol Study protocol. (PDF)

S1 Screenshot Example of the ACR program: Auditory instruction memory. (TIFF)

S2 Screenshot Example of the ACR program: Auditory time order judgement. (TIFF)

S3 Screenshot Example of the ACR program: Multiple object tracking. (TIFF)

S1 Video Example of cognitive remediation conditions. (MP4)
