# Correction: Cognitive function in multiple sclerosis improves with telerehabilitation: Results from a randomized controlled trial

**DOI:** 10.1371/journal.pone.0192317

**Published:** 2018-01-30

**Authors:** Leigh E. Charvet, Jie Yang, Michael T. Shaw, Kathleen Sherman, Lamia Haider, Jianjin Xu, Lauren B. Krupp

In Table 3, the mean baseline and end z-scores are incorrectly switched between treatment conditions. Please see the corrected Table 3 here.

**Table 3:** Composite score differences between conditions.

Treatment Condition*	Mean ± SD baseline z-score	Mean ± SD end z-score	Mean ± SD change z-score
ACR	-0.86 ± 0.77	-0.60 ± 0.85	0.25 ± 0.45
Active Control	-0.77 ± 0.73	-0.68 ± 0.76	0.09 ± 0.37

*5 subjects who dropped out the study did not have end z-scores and hence were not included in calculating end z-score and change in z-score.
