# Supplementary material for: Cognitive function in multiple sclerosis improves with telerehabilitation: Results from a randomized controlled trial
Source: PLoS One. 2017 May 11;12(5):e0177177. doi: 10.1371/journal.pone.0177177 (PMC5426671; doi:10.1371/journal.pone.0177177)
Supplement: S1 Protocol — (PDF) [file pone.0177177.s003.pdf]

MANUAL OF PROCEDURES

Interventions to Improve Cognitive Function in Patients with MS

A double-blind, randomized, controlled clinical trial to assess the efficacy of plasticity-based, adaptive, computerized cognitive remediation ("PACR") compared to an active control (ordinary computer games) in 136 adults with multiple sclerosis (MS).

Version 1.1
October 8, 2013
Updated December 31, 2014

CONFIDENTIAL

Interventions to Improve Cognitive Function in Patients with MS

A double-blind, randomized, controlled clinical trial to assess the efficacy of plasticity-based, adaptive, computerized cognitive remediation ("PACR") compared to an active control (ordinary computer games) in 136 adults with multiple sclerosis.

MANUAL OF PROCEDURES

Table of Contents

	Page #
1. Introduction	4
1.1. Study Rationale	4
1.2. Background	7
1.3. Preliminary Data	8
2. Specific Aims	11
3. Outcome Measures	11
3.1. PACR Program-Based Assessments	11
3.2. Neuropsychological Measures	11
3.3. Functional Measure	12
3.4. Interview-Based Rating	13
3.5. Participant Self-Report	13
4. Study Population	13
4.1.Number of Subjects	13
4.2.Participant Recruitment	13
4.3. Screening: Eligibility Criteria	13
4.4.Randomization	14
4.5.Reasons for Exclusion	14
5. Study Design	14
5.1 Overview of Study Flow	14
5.2 Blinding	14
5.3 Study Program Use	14
5.3a PACR Program	15
5.3b Active Control Program	16

6. Statistical Overview 16
6.1 Power & Sample Size 17
6.2 Testing the Primary Hypothesis 17
6.3 Interim Analysis 17

7. Facilities Available 18
7.1 Overview & Administration 18
7.2 Research Facilities 18
7.3 Clinical 18

1. INTRODUCTION

Cognitive impairment remains a major disability for individuals with multiple sclerosis (MS). The primary objective of this study is to evaluate the efficacy for treating MS-associated cognitive deficits using a unique computer-based plasticity-based and adaptive cognitive remediation treatment (PACR) compared to a computer-based control. This novel cognitive remediation approach has led to striking improvements in cognitive functioning in other disorders (schizophrenia, traumatic brain injury, aging, and dementia) but has never been applied to individuals with MS.

1.1 STUDY RATIONALE

RELEVANCE TO MULTIPLE SCLEROSIS

MS-associated cognitive impairment has adverse effects on many aspects of quality of life including impaired occupational and social functioning and increased caregiver burden. The proposed research is a direct response to the unmet need for effective treatment of cognition in MS.

The research will be the largest trial to date of cognitive remediation in this disease, has a randomized blinded design with an active control. The intervention is home-based and will allow wide patient access. Demonstrating a clinically meaningful treatment outcome would provide a new standard of care in MS. This immediately available program has the potential for increasing productivity at home and at work and enhancing quality of life, effects which could transform the lives of people with MS.

PACR program is a novel and innovative program with potential to help patients with MS
PACR is a program currently registered with the FDA to pursue regulatory approval, with the goal to become the first cognitive remediation program approved as medical device. A trial of PACR in individuals with MS is warranted because: 1) it is an innovative therapeutic approach that differs from other currently available treatments; 2) it is designed to target learning that is accompanied by neural reorganization or restoration; 3) it has a practical and novel delivery approach with a web-based implementation that can reach individuals with MS at any location; and 4) there is a significant body of randomized, controlled trial (RCT) data demonstrating that PACR improves cognitive and real-world function in people with a range of conditions and with forms of cognitive impairment similar to what is seen in MS.

1. PACR offers an innovative therapeutic approach by targeting procedural or skill-based learning: Most currently applied cognitive remediation approaches rely on the use of declarative memory in their implementation of training strategies. Unfortunately, declarative memory is typically involved in cognitive impairment. For example, when a patient is taught how to use a memory book, that patient must rely on their declarative memory to learn and then recall the steps required to use the memory book correctly [51]. Therefore, the patient is asked to rely on their deficient memory system for benefit.

In contrast, PACR is designed to enhance specific elements underlying cognitive function through extensive repetition of learning exercises. Task performance is over-learned through repetitive and rewarded practice. This practice-based skill learning depends on procedural memory. Procedural or skill-based memory typically remains intact in a variety of conditions marked by impaired declarative memory and other cognitive impairments [52-54], including MS [11].

PACR consists of exercises that continuously challenge basic processing skills, incorporating the critical elements thought to drive optimal learning [55-61]:

- A speeded schedule: driving the brain to process stimuli over brief periods of time, with extensive repetition of practice trials;
- Engagement: using attractive stimuli and high motivation for correct performance (correct trials are rewarded with points delivered through virtual shopping experiences and animations);
- Adaptivity: using adaptive tracking methods to continuously adjust the task to the sensory and cognitive capabilities of the individual participant (locking an individual's performance to 80-90% from trial to trial and across sessions to ensure that the exercises become more challenging for a specific individual's rate of learning at any given point in time); and
- Generalization: choosing stimuli to create exercises in which improvement will generalize broadly to other situations and real-world performance.

As shown in Figure 1, the exercises are based on the theory that an individual can restore degraded abilities through intensive procedural learning, leading to improved encoding of naturalistic information, resulting in improvements in cognitive functions based on the quality of that incoming information.

Figure 1. PACR Overview

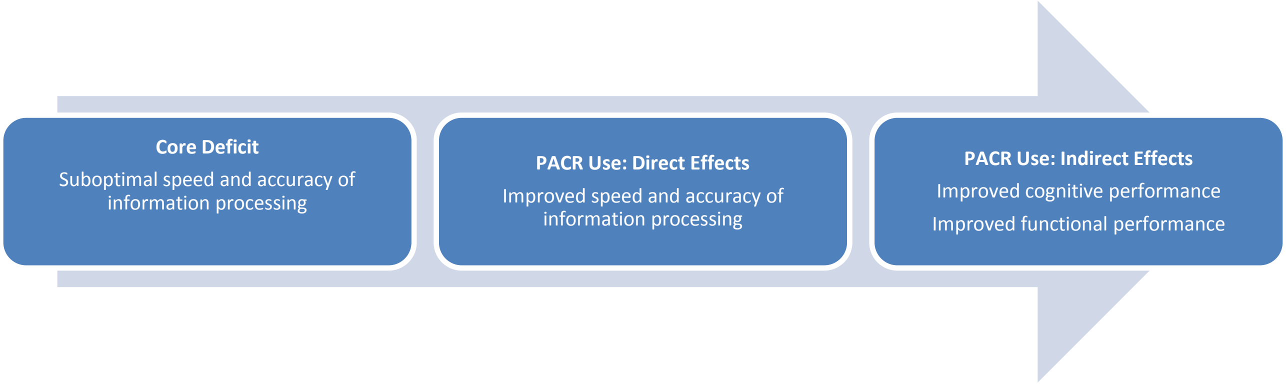

2. PACR is designed to target learning accompanied by neural reorganization or restoration: A large body of literature supports that, with appropriate forms of progressive learning, plastic changes in the adult brain can result in the acquisition or improvements in areas including: perceptual abilities [62-63]; motor response abilities [64-65]; attention system control [66]; processing speed [67-68]; and executive control abilities [69]. PACR stimuli and exercises have been designed to target these plasticity-based changes [51, 57, 70-71]. For example, a task may require auditory discrimination between two confusable syllables. Initially, the sounds are enhanced and slowed. This exaggeration is then gradually reduced across training while the individual continues to improve on this skill, with the goal of driving changes in the neural substrates of auditory discrimination and temporal response.

Studies of PACR in other conditions have documented changes that may suggest neural plasticity-based changes following program use [7, 21]. An fMRI study following PACR use in normal aging adults showed a greater activation in associated neural systems in the training group [72]. Task improvements correlated with increases in activation in the right inferior frontal gyrus and the precentral gyrus. An electroencephalographic (EEG) study [73] showed significant changes on visual evoked potentials in the N1 response over visual cortex. The magnitude of this change correlated with improvement in visual working memory. In a sample of 12 patients with mild cognitive impairment (MCI), pre- and post-training showed a significant difference within the left hippocampus as measured with fMRI [21]. In a

trial of PACR in adults with schizophrenia, cognitive improvement was accompanied by an increase in serum levels of brain-derived neurotrophic factor to levels similar to healthy controls, suggesting that physiological changes accompanied the cognitive changes [74].

3. PACR offers a novel and practical delivery approach: PACR is delivered over the web to any internet-connected computer, while providing continuous documentation of participants' usage and progress to a secure central server for ongoing review and analysis. In contrast to previous cognitive remediation approaches requiring a patient visit with 1:1 sessions with a trained clinician, web-based delivery can be offered to more participants at reduced cost. It is also likely to improve overall clinical outcomes by treating more patients in need than is possible with existing techniques, with the delivery of more therapy to each patient.

4. PACR has demonstrated efficacy: Multiple randomized controlled trials (RCTs) have demonstrated that PACR improves cognitive and functional abilities in diverse patient populations with cognitive impairment that is similar in type and range as that seen in MS. Endpoints have included both neuropsychological assessments of memory and cognition and real-world function. Currently, large multi-center trials of PACR are underway in the areas of mild TBI and schizophrenia[23-24].

Normal aging: In the largest RCT of computerized cognitive training programs to date, 487 cognitively normal adults aged ≥65 years were randomized into treatment and active control (educational content) group [75]. Each group completed 40 hours of training (1 hour/day, 5 days/week for 8 weeks total). Results demonstrated: 1) training-to-the-task improvement as expected, 2) generalization of improvement to a composite of untrained, standardized neuropsychological assessments, and 3) generalization of improvement to a measure of everyday cognition. Three additional randomized controlled trials have been completed in normal aging (n=91, n=126, n=105) [76-78]. These studies demonstrated that PACR exercises targeting useful field of vision significantly improved the timed instrumental activities of daily living (TIADL task; the outcome for the first two trials) and reaction time in a driving simulator and on-road driving performance (the 3rd trial) [78].

Mild Cognitive Impairment: A RCT of 47 participants with MCI compared treatment to active control (computer use) with 40 hours of training (90 minutes /day, 5 days/week for 5 weeks total) and showed a strong effect on pre-post performance on a cognitive battery and a trend towards improvement on delayed memory [20].

Schizophrenia: An initial RCT in schizophrenia included 55 participants. Treatment was compared to an active control (computer games), with both conditions completing 110 hours of training (1 hour/day, 5 days/week for 22 weeks total). An interim assessment (50 hours of training) showed a significant improvement in global cognition with particular large effects on verbal learning and working memory [8]. Continuation for the remaining 60 hours of program use showed further growth in the overall effect size, and then a general maintenance of the cognitive improvement after a six-month no-contact follow-up period [19]. The effect of PACR on global cognitive function in schizophrenia was recently confirmed and extended in a multi-site replication study [79-80]. This multi-site study enrolled 53 participants with schizophrenia randomized into treatment and active control (computer games) groups, with results similar to the original findings. An additional RCT with 39 participants comparing PACR to an alternate therapy (CogPack) showed significant improvements in verbal learning and memory in the PACR group and normalization in the PACR group only on a standard electroencephalographic measure of the integrity of early brain information processing (sensory gating ratio)[81].

HIV: A recent controlled pilot study was completed with 46 middle-aged and older adults with HIV [22]. Approximately half (n=22) were assigned to complete 10 hours of PACR games targeting speeded visual processing compared to the control group that received no contact during the study period. Despite the relatively short period of intervention, the PACR group showed significant improvements compared to the controls on the TIADL task, a speeded visual task (Useful Field of Vision) and finger tapping.

Summary of previous studies: In aggregate, these findings support PACR efficacy across a variety of patient groups and hold two main implications for work in MS: 1) PACR is appropriate for MS: across the spectrum of cognitive function, ranging from high-functioning individuals undergoing normal cognitive aging to low-function individuals with schizophrenia, the PACR exercises are learnable and usable by individuals in an in-home setting; 2) PACR is likely to be effective in MS-related cognitive impairment: across several indications with distinct underlying causes there is substantial evidence that PACR drives changes in function that generalize to both neuropsychological and real-world measures of function.

1.2 BACKGROUND

Cognitive impairment is a significant unmet treatment need in MS

Multiple Sclerosis (MS) is characterized by demyelinating lesions throughout the central nervous system and is the most common progressive neurological disorder to occur in working-age adults [28]. Cognitive impairment, estimated to occur in more than half of all patients, can be independent from disease progression [29]. The most common deficits are in the areas of information processing, attention, and new learning [9-10, 30]. These deficits often strike individuals during their key years of productivity and are associated with significantly compromised quality of life and increased caregiver burden [31].

Unfortunately, pharmacological approaches have not shown consistent treatment benefit [32-33]. Disease-modifying medications may slow the progression of deficits, but studies have had varied findings and methodological challenges [27, 34-37]. Symptomatic medication approaches (e.g., cholinesterase inhibitors such as donepezil [16, 26] or amphetamines [12, 14]) have been of limited use, with initial positive findings unconfirmed in larger trials [14, 26]. Most recently, a subgroup analysis of more impaired patients in a trial of L-amphetamine showed a positive signal for cognitive enhancement [14-15]. However, even if proven to be effective for some, the use of amphetamines may have considerable treatment limitations, including the potential for abuse.

Cognitive remediation is a promising treatment option

Cognitive remediation (also referred to as cognitive rehabilitation or cognitive training) holds particular promise because it is safe and noninvasive and works to restore functional abilities [38-39]). Despite its promise, conclusions concerning cognitive remediation approaches have been limited due to methodological problems. Relatively few studies of cognitive remediation programs have included adequate sample sizes and/or controlled designs. For instance, a recent review noted 112 studies of cognitive remediation in TBI and stroke, with only 14 meeting criteria for consideration as Class I level of evidence [38]. Similarly, reviews of cognitive remediation in MS have noted few trials to have used rigorous study design [40-41].

A second major limitation for cognitive remediation has been the variability of approaches to cognitive remediation programs. Traditionally, cognitive remediation has been delivered through the drill-and-practice of information and learning strategies to target specific cognitive skills (e.g., memory techniques) and require 1:1 sessions with a trained clinician. More recently, technological advances have led to computerized programs. Computer-based programs offer several recommended advantages, including the ability to provide more intensive therapy [42] and the capability to adapt program administration to meet individual levels of function and performance [43]. Computer-based delivery often does not require as much clinician time, and can provide the option of delivery to remote (e.g., home-based) locations.

The few previous studies of cognitive remediation in MS [40-41] have shown clear signals of a positive treatment effect [40-42, 44-50]. In the largest trial to date, Shatil and colleagues completed a controlled trial with a computer-based training program (CogniFit Personal Coach) in 107 Israeli patients with MS for 12 weeks [44]. Results showed rapid recruitment and, despite no further contact after baseline, strong unprompted adherence to the program (71.2% used the program and 57.6% completed the majority of their sessions). The treated group improved on more cognitive measures than the control group, with significant changes on measures of general memory, and verbal and visual working memory. Unfortunately, the study was not randomized and did not include an active control condition. However, findings support the potential benefit of a computer-based cognitive remediation program for individuals with MS to use from home.

Reviewers have uniformly concluded that future trials should include sufficient sample sizes and study designs that include an active control condition [39, 43]. Ultimately, benefits from the interventions should generalize beyond the training task and/or single cognitive domain of function [49]. It is also recommended that programs to be evaluated for use in MS should first have efficacy demonstrated in other conditions [40].

The proposed trial is designed to overcome the limitations of prior cognitive remediation trials in MS and incorporates the reviewer recommendations. This study will have an adequate sample size, be double-blinded and randomized, and include an active control condition. The intervention, PACR, is based on recent advances in both neuroscience and technology. It offers a cutting-edge program design with web-based delivery. PACR also has the most empirical support for any cognitive remediation program developed to date.

1.3 PRELIMINARY DATA

The goals of our preliminary data collection were to: 1) estimate the ***recruitment rate*** based on the ability to identify eligible participants; 2) assess PACR program ***compliance*** in enrolled participants; and 3) measure for a signal of benefit in ***treatment outcomes*** (PACR program performance, participant ratings, and neuropsychological measures).

1. Recruitment Rate: Potential participants were identified through clinician referral, without any advertising or other outreach. MS patients were considered eligible if their treating neurologist judged that cognitive impairments were a concern; they were interested in completing computer-based games that may improve their cognitive functioning; they had sufficient motor dexterity and visual ability to operate a laptop computer; and they had internet access at home.

Results: Our goal for the feasibility study was to recruit ten participants. However, we met with significant interest from the patients in our clinic, identifying 32 eligible participants at a rate of two per week, over 16 weeks. Program enrollment was limited by the availability of computers and study staff.

Ten (10) participants have completed the PACR program to date and their outcomes are reported below. These participants were three (3) men and seven (7) women, ranging in age from 27 to 64 years (mean of 49 years). Six had a diagnosis of relapsing-remitting MS and four had a diagnosis of primary progressive MS. EDSS scores ranged from 1 to 6.5, with a mean of 3.8.

2. PACR Program Compliance: At ***baseline,*** participants visited the clinical research unit and completed a brief neuropsychological evaluation. Participants received a laptop computer and a set of noise-cancelling headphones, and were instructed on how to access the PACR program. During the ***remote (home-based) treatment period***, participants were asked to complete the study games for 1 hour/day, 5 days/week, for a total of 40 hours over eight weeks. The 40-hour target was based on previous feasibility studies prior to larger controlled trials other conditions [8, 25]. Compliance data were collected remotely. Participants returned after eight weeks for the ***study end visit***, complete the follow-up evaluation and return study equipment.

**Table 1: Total Program Hours**	
101	40
102	59+
103	42
104	51
105	50
106	31
107	73+
108	40
109	42
110	42
**MEAN**	**47**

Results: As shown in **Table 1**, all participants were close to the target of 40 hours. Nine of the ten completed or exceeded the target amount of hours. The remaining participant (#106) completed 31 hours. Two participants (102, 107) requested to continue sessions on their home computer after the eight week assessment.

3a. Treatment Outcome- PACR Performance: In addition to tracking compliance, the PACR system provides measurement for a participants' progress over time, as a check for successful training to the task. Performances are compared to a large normative database, provided by the program developers, and each participant is assigned a percentile to their current skill level. Change in percentile ranking between the participant's initial and best performance serves as an indicator of skill progression within the program.

Results: As shown in **Figure 2**, all participants showed improvement in their percentile ranking (combined across tasks), with the average group gain of 30.9 points, indicating marked improvement on the program tasks.

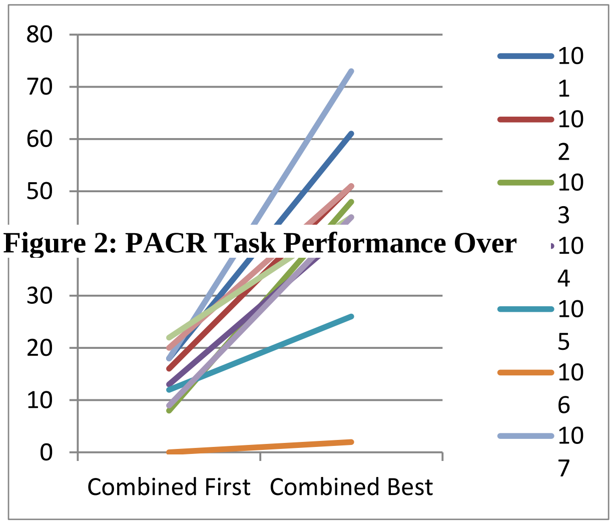

Figure 2: PACR Task Performance Over

3b. Treatment Outcome- Participant Ratings: At the study end visit, participants reported their impression of their cognitive functioning (memory and global cognition) as a result of

completing the PACR program. A study staff member also completed the Clinician Interview-Based Impression of Change (CIBIC) rating with both the participant and an informant when available (n=4, either a spouse or other family member).

Results: The majority of the completed participants reported improvement. Seven of the 10 participants reported improved general cognition, and six reported improved memory. Similarly, seven of the 10 received ratings of at least mild improvement on the CIBIC. No participant reported nor received a rating of decline.

Participants and/or their informants were also provided with space to add any written comments along with their ratings and these were overwhelmingly positive.

Participant comments included the following: *"I think I am more comfortable with my deficits and my emotional reaction to forgetfulness has improved along with my cognition."; "Multitasking has improved."*; *"Using the program improved attention span and concentration."; "At MS meeting remembered to say what I need to say after the person before me is done speaking, I usually forget by that point." ;* **"***I have an easier time remembering conversations I've had and little things I need to do."; "I have noticed some noticeable cognition, conversation suggestions, memory recollection. Seems to be better. Much more cognizant."*

Table 2. Neuropsychological Performance			
Participant	**Composite Cognitive z Score**		
	**Baseline**	**Study End**	**Change Score**
101	-.51	.13	.64
102	-1.01	-1.03	-.02
103	-.11	.34	.45
104	-1.20	-1.03	.17
105	-2.36	-1.75	.61
106	-.54	-.31	.23
107	-2.58	-1.88	.70
108	.46	.79	.33
109	.09	.07	.02
110	.23	.39	.16
**MEAN**	**-.75**	**-.43**	**.33**

Comments from the informants included: *"The skills offered here helped her."; "I think yes overall improvement. She went after it diligently." "Remembering the date now several times a day and is able to repeat it and get it right." "Remembers little things that she would have forgotten before."*

3c. Treatment Outcome – Neuropsychological Measures: A composite score was derived from four neuropsychological measures: California Verbal Learning Test-Second Edition (CVLT II)[82] total learning score (verbal learning), Brief Visuospatial Memory Test-Revised (BVRT-R) [17] total learning (visual learning), the Symbol Digit Modalities Test (SDMT) [1-2, 5] total score (information processing), and Delis-Kaplan Executive Function System Trail Making Test (DKEFS Trail Making Test) [18] Letter/Number Sequence trial (executive functioning). These measures have reliable alternate forms and are both sensitive to the impairments typically seen in patients with MS, and were comparable to the composite outcomes used in evaluation of the PACR program in other conditions. Participant scores were transformed to z scores derived from published normative data for each of the four measures, and then averaged for one composite score. Change scores were then calculated between baseline and study end.

Results: As shown in **Table 3**, baseline, participants' cognitive composite scores ranged from z=-2.8 to z=.46, with a mean of z=-.75, indicating a range of cognitive functioning across the sample trending

towards low average to mild impairment as a group. After completing the PACR program, all but one participant showed improvement, with a mean increase of z=.33.

Summary of Preliminary Data: Our feasibility study indicates that eligible and interested participants can be readily identified. PACR program compliance rate is very high. A positive signal for improvement was identified through improvement on the PACR program exercise, participant ratings, and standardized cognitive measures.

2. SPECIFIC AIMS

- Specific Aim 1 – Evaluate the effect of PACR on generalized cognitive and functional performance:
  Co-primary outcome measures will be: 1) a composite derived from a standardized battery of neuropsychological tests, and 2) a timed measure based on direct observation of functional performance.

- Specific Aim 2 – Identify specific predictors of response to guide future use:
  Covariates will include baseline cognitive status, PACR program compliance, PACR task improvement, and participant-reported outcomes.

PACR program is a novel and innovative program with potential to help patients with MS
PACR is a program currently registered with the FDA to pursue regulatory approval, with the goal to become the first cognitive remediation program approved as medical device. A trial of PACR in individuals with MS is warranted because: 1) it is an innovative therapeutic approach that differs from other currently available treatments; 2) it is designed to target learning that is accompanied by neural reorganization or restoration; 3) it has a practical and novel delivery approach with a web-based implementation that can reach individuals with MS at any location; and 4) there is a significant body of randomized, controlled trial (RCT) data demonstrating that PACR improves cognitive and real-world function in people with a range of conditions and with forms of cognitive impairment similar to what is seen in MS.

3. OUTCOME MEASURES

3.1 PACR Program-based assessments: As in our feasibility trial, we will measure: *1) Compliance*: total number of hours of program use; and 2) *PACR performance*: participants' progress will be measured from built-in assessments and compared to a large normative database provided by the program developers. We expect large improvements in the treatment group on these assessments because they have directly practiced these tasks. Participants failing to make progress on exercise-based assessments may represent a subpopulation not treatable with this program, and participants making strong progress may represent a subpopulation particularly amenable to treatment.

3.2 *Neuropsychological Measures: The complete evaluation as shown in **Table 4** will be performed at baseline and study end, with the exception of the Wide Range Achievement Test- 3rd Edition Reading Test (WRAT-3) [84], which will be administered only once at screening/baseline. Tests will include the Symbol Digit Modalities Test [1-2, 5], Paced Auditory Serial Addition Test (PASAT) [5-6], Wechsler Adult Intelligence Scale-Fourth Edition (WAIS-IV) Digit Span and Letter/Number Sequencing [89], Selective*

Reminding Test [5, 90-91], Brief Visuospatial Memory Test [17], DKEFS Trail Making Test [18] and 9-Hole Pegboard Test [92-93].

Alternate forms of each test will be used to mitigate test-retest effects. Participants who stop program use will be scheduled for assessment based at three months from their consent date. Participants seeking to withdraw from the study will, with their consent, be scheduled for a final assessment at the time of their withdrawal.

*We will employ a single composite cognitive measure as a co-primary outcome that will be derived from four scores from the test battery, as shown in **Table 4**. These four measures include: 1) PASAT total score (), 2) SRT total trial learning, 3) BVMT-R total trial learning, and 4) DKEFS Trails composite number and letter sequence score. These measures were selected due to their: 1) sensitivity to the change in studies of other interventions targeting cognitive processing in MS [12, 14-16], 2) reasonable test-retest stability and minimal practice effects, and 3) plausibly to reflect PACR-related improvements (based on prior studies and our feasibility trial).*

The summary score (see Section 6) from the Timed Instrumental Activities Daily Living Task will be a co-primary functional outcome.

Table 4. Study Evaluation Procedures for Screening, Baseline and Study End		
Neuropsychological Measures (administered by blinded psychometrician)		
Test	*Domain*	*Study Outcome*
WRAT-3 Reading	*Premorbid IQ Estimate*	*Randomization (Screening Only)*
SDMT	*Screening, Level of Impairment*	*Randomization*
PASAT	*Information Processing*	*Co-Primary (total score*)*
WAIS-IV Digit Span-Forward	*Information Processing*	*Co-Primary (total score*)*
WAIS-IV Letter Number Sequence	*Information Processing*	*Co-Primary (total score*)*
SRT	*Verbal Learning*	*Co-Primary (total learning trials*)*
BVMT-R	*Visual Learning*	*Co-Primary (total learning trials*)*
DKEFS Trail Making Test	*Executive Functioning*	*Co-Primary (number and letter trial*)*
WAIS-IV Digit Span-Forward	*Information Processing*	*Secondary*
9-Hole Pegboard	*Motor*	*Secondary*
Functional Measure (administered by blinded psychometrician)		
Timed Instrumental Activities of Daily Living task (TIADL)		*Co-Primary*
Interview-Based Rating (administered by study nurse practitioner)		
Cognitive Assessment Interview (CAI; with informant input)		*Secondary*
Self-Report Measures		
Participant-Reported Outcomes (with informant input)		*Secondary*
MS Neuropsychological Questionnaire (MSNQ)		*Secondary*
Ruff Neurobehavioral Inventory (RNBI)		*Secondary*
BAMA (with informant input)		*Secondary*
ECOG (Test of Everyday Cognition) (with informant input)		*Secondary*
BDI-Fast Screen for medical patients		*Secondary*
CMDI Self-Report Scale		*Secondary*
Fatigue Severity Scale (FSS)		*Secondary*

Modified Fatigue Impact Scale (MFIS)	*Secondary*
Positive and Negative Affect Schedule (PANAS-SF)	*Secondary*
Benefit Finding (with informant input)	*Secondary*
Post-traumatic growth inventory (with informant input)	*Secondary*
** six representative scores will be compiled into a composite z score for the co-primary outcome measure*	

3.3 Functional Measure: The Timed Instrumental Activities of Daily Living task (TIADL) is a test of real-world performance. The TIADL task is designed to be sensitive to mild cognitive impairment and has published test-retest reliability [94-95]. TIADL completion time has demonstrated sensitivity in studies of MS [96] as well as an outcome in PACR trials in normal aging [76-77], mild cognitive impairment [97], and HIV [22]. TIADL scoring rules result in a single composite outcome measure for use as the functional co-primary outcome measure in this study [98]. Completion time is measured in five key domains represented by 10 common daily tasks: information identification finding a telephone number), finances (making change), food (reading the first three ingredients on a can of food), and shopping (finding two items on a shelf), and medication management (reading the directions on a medicine bottle).

3.4 Interview-Based Rating: A blinded study staff member will administer the Cognitive Assessment Interview (CAI) [99]. The interview includes informant input when available and provides a clinician interview-based rating for cognitive function on specific domains and a global impression.

3.5 Participant Self-Report*:* Program outcome ratings by the participant and their informant. Participants will also complete the MS Neuropsychological Questionnaire (MSNQ) [40, 100], the Ruff Neurobehavioral Inventory [101]. [101], the ECOG (participant and their informant),the BAMA (participant and their informant) , BDI-Fast Screen for medical patients, CMDI Self-Report Scale, Modified Fatigue Impact Scale (MFIS), Fatigue Severity Scale (FSS), the Positive and Negative Affect Schedule (PANAS-SF), Benefit Finding (participant and their informant), and the Post-traumatic growth inventory (participant and their informant).

4. STUDY POPULATION

The proposed study will be a double-blind, randomized trial of PACR versus active control (ordinary computer games), administered for 60 hours across three months in 136 individuals with MS.

4.1 Number of Subjects

A total of 136 subjects with MS will be enrolled over the course of the study.

4.2 Participant Recruitment:

Target enrollment will be five participants per month. The study will be advertised through the Stony Brook MSCCC clinic where over 1000 MS patients are seen annually. There is a growing waitlist for enrollment from our feasibility study. Other sources of recruitment include area neurologists, emails to patient lists, the local MS Quarterly Newsletter, written advertisements posted in the MS Center, local community newspapers, and the Stony Brook University Medical Center patient newsletter and website.

All participants will have access to the treatment program (either as active treatment, or at study end if in the control condition), as below. Participants will be reimbursed for the baseline and study end visits to assist with any costs they may incur (e.g., work absence, babysitting, travel, etc.).

4.3 Screening:
We will enroll participants with at least probable mild cognitive impairment, as determined by an SDMT score falling one standard deviation or below published age-reference normative data [1-2]. Otherwise, eligibility is purposely designed to be broad. Specific Aim 2 is designed to identify predictors of patients who may gain the most benefit from PACR use in the future.

Eligibility Criteria

Inclusion Criteria

- *Ages 18-70*
- *Definite MS Diagnosis, any subtype [83]*
- *Probable cognitive impairment as defined by a score ≤ 1 SD below the mean of normative data on the SDMT*
- *Concurrent medications to be kept constant over three months (as possible)*
- *No relapse or steroids in previous month*
- *Reading score on WRAT-3 of 37 or greater*
- *Visual, auditory and motor capacity to operate computer software, as judged by treating neurologist or study staff*
- *Willing to sign Agreement to Borrow Laptop*

Exclusion Criteria

- *History of mental retardation, pervasive developmental disorder or other neurological condition associated with cognitive impairment*
- *Primary psychiatric disorder that would influence ability to participate*
- *Other serious uncontrolled medical condition (e.g., cancer or acute myocardial infarction)*
- *Alcohol or other substance use disorder*
- *History of computer-based training manufactured by Posit Science*
- *Learned English language after 12 years of age*

4.4 Randomization: *At screening, evaluation will include two brief cognitive measures to be used for stratified randomization: 1) the Wide Range Achievement Test-3rd Edition (WRAT-3) [84], to serve as a general estimate of premorbid IQ, and 2) SDMT[1-2]. The SDMT has been demonstrated to be sensitive to the cognitive deficits associated with MS [85]. Here, the total score will be used as a general estimate of current level of cognitive impairment. Scores will be compared to published normative data and categorized according to whether they fall within one standard deviation from the normative mean; one, two or three or more standard deviations above; or one, two or three or more standard deviations below. Age will also be a stratification factor.*

4.5 Reasons for excluding participants during the trial: *Any participant who during the trial experiences a relapse that is sufficiently severe to interfere with daily functioning or to require steroids will be excluded.*

5. STUDY DESIGN

5.1 Overview of Study Flow:
The study flow is shown in **Figure 3 (pg 17)**. At the baseline visit participants will complete a *baseline evaluation* (see below) and receive *study program instruction.* They will receive study equipment (laptop and headphones; internet card if needed); be instructed on their assigned program; and access the program under clinical supervision. *Study Program Use* (see also below): Participants will be instructed to use their assigned program, in a quiet, distraction-free location of their choice. Study programs are to be used in one-hour sessions, with a goal of five sessions per weeks, for 12 weeks total

following randomization. Study End: Following final program use session, participants will return for their final study end evaluation. Study laptop and headphones will be returned at this time for reassignment.

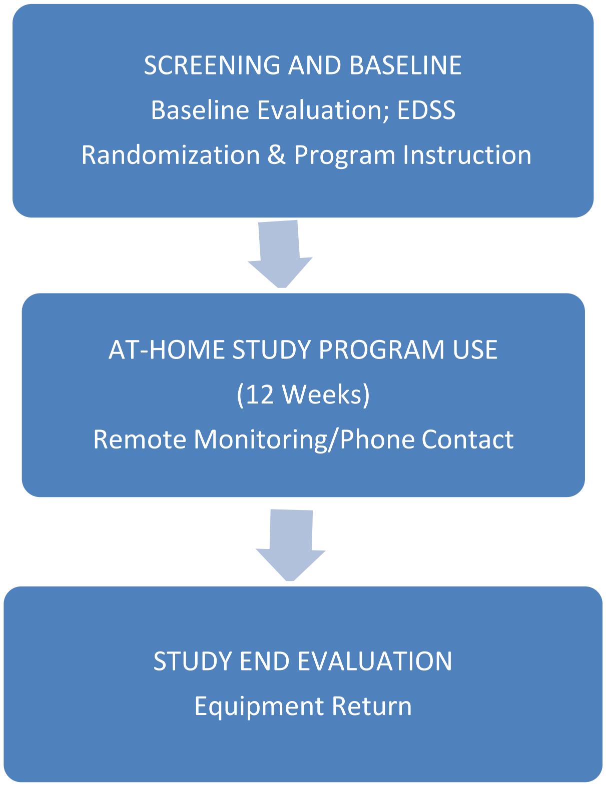

5.2 Blinding: The treatment condition and the control condition will be identified as “Treatment X” and “Treatment Y”. All participants will be instructed to complete computer games that may have cognitive benefit from home using the study-provided laptop. The Co-PI (neuropsychologist) and Clinical Coordinator will remain unblinded. The Clinical Coordinator will provide the randomization assignment (computer-generated), register participants to their respective computer programs, provide instruction, and monitor initial program use.

The PI and study psychometrician will remain blinded. Only the blinded study psychometrician will administer the baseline and study end cognitive testing and questionnaires. Data entry will be double-coded by a second blinded psychometrician. Blinded study staff will complete the clinician-based interview. At the end of the trial, study staff will evaluate the integrity of the blinding procedures.

5.3 Study Program Use
All participants will be loaned a study laptop for the duration of the program-use portion of the study; a subset of the computers will be equipped with a wireless internet card for any participant without home internet access. Participants will use their assigned computer-based intervention (referred to as “Treatment X” or “Treatment Y”) for one-hour sessions, with a goal of five sessions per week. Each participant will have phone contact at least once per week.

Figure 3. Study Overview
Sixty hours of use are anticipated over a three-month period (12 weeks). Participants who have not completed 60 hours of computer-based cognitive intervention within 12 weeks of the initiation of use will be offered the opportunity to continue program use for no more than two additional weeks. Participants will not be permitted to complete more than 70 total hours of program use before their study end evaluation.

5.3a PACR Program: To use PACR, the participant opens a standard web browser on a broadband connected computer and navigates to the PACR study website. The study web site serves simply as a gateway to the games and contains no information to identify it as a cognitive remediation program and does not have any reference to the program developers. The participant then logs into the PACR (using a study provided screen name and study identification number). A game-like experience begins, where

the participant is encouraged to earn points and in-game rewards to advance. To do so, the participant selects one of the cognitive exercises scheduled for the day, and performs that exercise for fifteen minutes. The exercise itself contains the core science stimuli and tasks. The exercises rely on procedural memory systems and performance is expected to improve with practice. Participants perform tens to hundreds of trials over the course of the fifteen-minute session, with each trial providing auditory and visual feedback and rewards to indicate if the trial was performed correctly or incorrectly. After each trial, the difficulty of the next trial is updated to ensure that within a session, the participant gets approximately 85% of trials correct. The scheduling mechanism ensures that a participant progresses through the exercises in a defined order, generally moving from more simple (early sensory processing) exercises to more complex (multimodal, cognitive control) exercises over the course of the three-month experience. At any point in time, the participant only has access to a subset (typically six) of these exercises, four of which are performed per day. Each exercise has specific criteria for completion or plateau performance, and after those criteria are met the exercise is removed from the active set and the next exercise is added to the active set. This mechanism provides ongoing novelty and engagement for the participant, while ensuring that the participant progresses smoothly through the complete set of exercises over the program use period.

There are a total of 13 cognitive modules in PACR (see **Appendix 1**). During each session, exercises from four modules are presented. The modules are completed in a set order, and when one is completed the next module is started. With 40 hours of use, nine of the ten completed participants from our feasibility trial finished the first four modules and were working on exercises from the following four. We expect that with 60 hours, participants in this study on average will work on exercises from the first ten modules, following six modules to completion.

All usage and progress data are encrypted and then transmitted to a central server. No personally identifiable information is stored on the server. On the server, the data are available for review through a secure web portal, used to regularly check on usage and progress of each active participant to customize their weekly phone contact in order to provide helpful guidance and coaching as needed.

5.3b Active Control Program (Ordinary Computer Games): The active control program is composed of 13 ordinary computer games (see also **Appendix 1**) matched to the PACR condition in overall procedures and program use intensity [20, 75, 86-87]. This condition is designed to be a face-valid approach to cognitive remediation in order to maintain the participants' blinding. The control condition is also designed to account for nonspecific treatment effects, including placebo response, interactions with research personnel, and experience with computers and computer-related activities, and any halo or expectation effect on study assessments.

To use the active control, a participant launches a local game controller on their desktop, and then logs on to a user screen. The local application then allows the participant to select from a number of ordinary computer games installed on the computer (e.g., word search, puzzles, etc.). The games are chosen for their face-validity as cognitively challenging activities. Participants are instructed to play four games for 15 minutes each, according to a set rotational sequence.

Participants will be asked to connect to the internet at least once per day, and program usage data will be remotely monitored with the secure software program Track4Win [88]. As with the PACR condition, the secure web portal will be used to regularly monitor participants' program usage. These data will be used to customize their weekly phone contact. This ensures that the PACR and active control groups are matched for social contact and encouragement from study staff.

6. STATISTICAL OVERVIEW/SAMPLE SIZE AND ANALYSIS PLAN

As displayed in **Figure 4**, this is a two-group, randomized controlled trial with a parallel group design. Subjects will be stratified by participants' ages (18- <35, 35-50, and >50), WRAT-3 Reading Score for premorbid IQ estimate obtained at the baseline (-1 - -1.99, -2 - -2.99, and below -3) , and SDMT for current level of cognitive impairment obtained at the baseline (-1 - -1.99, -2 - -2.99, and below -3). This results in 9 strata. Within each stratum, subjects will be randomized 1:1 to either intervention or control group. Furthermore, in order to ensure similar group assignments within each stratum, blocked randomization is used with random permuted block size of 4 and 6. All eligible patients who are registered and randomized will be included in the analyses, consistent with the intent-to-treat principle. There are two co-primary quantitative outcomes; a significant finding in either quantitative outcome will claim efficacy for PACR so all of the study's type I error rate of 0.05 will be "spent" in testing efficacy Hypothesis 1, and this can be done using 0.05 type I error rate for each outcome.

<u>**Calculation of Primary Outcome Measures**</u>

1. **Neuropsychological Composite:**

To evaluate change on the composite cognitive measure co-primary outcome, raw scores will be transformed to z scores to provide a standard metric. The z scores will be calculated using means and standard deviations of the total group performance at baseline to calculate z scores for both baseline and follow-up measures.

1a. The mean of the group-derived z score for the composite neuropsychological test variables will then be computed for each subject at baseline and follow-up.

Representative raw scores from the following tests will be used in the composite:

- BVMT-R Total Learning: "bvmt_total_raw"
- SRT Total Learning: "srt_total_raw"
- Digit Span Backwards: "digitspan_backward"
- Letter-Number Sequence: "lett_num_seq_raw"
- PASAT- 2 score: "pasat2_raw"
- DKEFS Trails Letter-Number Composite*: "LettNumCombo_DKEFS=dkefs_trails_2 + dkefs_trails_3"

*<u>Database note</u>: this DKEFS Trails Letter-Number Composite score is derived by summing two raw scores in database; also note time score needs to be reverse scored, so higher (slower) score indicates poorer performance (in order to be consistent with the other measures for the composite, where lower score indicates poorer performance).

1b. The mean of the z scores for each measure will be calculated for each participant at baseline and follow-up. Next, the difference between these two means (mean composite z score at time 2 – mean composite z score at time 1) is to be calculated.

1c. The difference between these two scores (change from baseline) will define the primary outcome for the neuropsychological composite measure.

2. TIADLS:

This measure consists of 10 items. Items 1-9 are timed, with scores from 0 to 120 or 180 seconds,* depending on task. Each measure is then given an error score of 1, 2, or 3 (representing no error, minor error or major error/failure to complete in time; note that item 10 only has an error score).

2a. To score: for items 1-9, each raw time score is multiplied by its error score. For example, for item 1, the score would be (tiadl_t1) *(tiadl_a1). Total score for a 120 second item is 120 x 3 =360, and total score for a180 second item is 180 x 3=540. Item 10 is left as a score of 1,2 or 3 (time is not part of score for item 10).

2b. Items are then converted to group-based z scores to provide a standard metric and consistent weighting of each item in the total. First, means and standard deviations are calculated for the entire group at baseline for each item's score. These means and standard deviations will be used to calculate both baseline and follow-up z scores. In this process, item 10 is also converted to a z score, and therefore provides equal item weighting.

2c. The total score at each time point will be the total of item z scores (1-10), providing a total baseline score and a total follow-up score.

2d. The difference between these two totaled scores (change from baseline) will define the primary outcome for the neuropsychological composite measure. Note here that the scores are reversed because they represent

*Database note: Due to differences in data recording, in the current database, some raw scores need to be capped at the time limit as part of the data cleaning process. Currently, at times total time, even if over the time limit, have been entered. As an example, if a time was entered as 137 seconds for a 120 second time-limit item, the score needs to be capped to be counted as 120 (ie they reached the time limit). Any score over 120 and 180 seconds respectively (depending on item) should be counted as 120 or 180 with error score of 3 to indicate incomplete). So, the total time score possible on any item would be either 120 or 180 seconds (depending on item) x 3 (for incomplete within time allowed).

<u>Analytic Approach</u>

Figure 4 : Diagram of study design	
Elapsed time since randomization	
Months:	0 3
PACR:	**A**+++++++**B**
**Control:**	C**oooooo**D

Referring to Figure 4, following balanced randomization, Month 0 study entry is at times A and C. The PACR group is treated from A-B and the Control group receives their intervention from C-D. Inferential analyses will be performed using random effects regression models in SAS/STAT PROC MIXED.

This section provides a brief overview of statistical topics, e.g., sample size and tests of the primary hypotheses. A *Statistical Analysis Plan*, to be written before the analysis is implemented, will include details about these and other statistical topics such as: testing secondary hypotheses about real-time, within-session results for compliance and performance; evaluating sensitivity of hypothesis tests to missing data (use Multiple Imputation [102]; and selecting covariates for inclusion in mixed effect regression models.

6.1 Power and sample size: There are two, primary, continuous outcomes; this calculation concerns the Month 3 between-group comparison to test Hypothesis 1, which is the primary efficacy hypothesis. To claim efficacy for PACR, tests of either outcomes must reject the null hypothesis of equal means, so that both tests can use 0.05 type I error rate for a 2-sided test. Because of the absence of a priori knowledge about the effect size within each stratum, we assume each stratum has similar effect size and variability. Therefore, a standard t-test calculation can be used to compute power *vs*. effect size for the proposed sample size of n = 68 per group, which yields n = 65 per group with 5% missing at Month 3. (However,

the actual data analysis, by mixed effect regression in SAS/STAT PROC MIXED, is more flexible than a t-test and has higher power since it makes more effective use of baseline data (times A and C). Based on clinical input, and from our previous studies in TBI and schizophrenia, we estimate standardized effect size (Cohen's d) of 0.5 to be the "Smallest Clinically Meaningful Effect." In PASS12 sample size software, this yields 81% power.

6.2 Testing the primary hypotheses: Comparisons based on randomization can be tested in PROC MIXED using a model with a random effect for participant within group (PACR *vs*. Control), and terms for participant, time point (0 = baseline, 1= Month 3),intervention (PACR, Control). Also the three stratification factors, age, WRAT-3 reading score and SDMT score will be included in the model as covariates. With no missing data, all participants will have two records. All collected variables will be analyzed in a descriptive manner first. Patients' characteristics will be compared between two study arms through Chi-square tests (for categorical variables) or t-test (for continuous variables) to confirm the performance of randomization scheme.

Hypothesis 1: At Month 3, Hypothesis 1 asserts PACR is more effective than the control (video games) (B better than D) on either co-primary outcome measures. Stratified t-test will be used to test this hypothesis first. Furthermore, linear mixed model will be also fitted to test if the improvement over time between two study arms is significantly different or not. The significant interaction between group and time, or the contrast (B-A)-(D-C) will suggest that PACR and control have significantly different effect. All null hypotheses are that a term is 0 and all tests use 0.05 type I error rate. The model assumptions for linear mixed model such as linearity, normality will be assessed and data transformation may be needed to make these assumptions met.

Hypothesis 2: Several covariates (baseline function, overall task compliance (number of completed sessions), improvement on study tasks (increased percentile scores), and patient-reported outcomes predict PACR benefit. Month 3 PACR benefit is the interaction (B-A)-(D-C). A covariate z can independently predict these effects if that covariate is a significant factor in the linear mixed model with either primary outcome as the response variable. *Clinical features such as presence/absence and type of DMT, MS subtype, and disease duration will also be examined for the relation to treatment effect. However, these are not expected to be strongly associated with treatment outcome.*The predictive accuracy of the final model will be unbiasedly validated using bootstrapping (REF).

7. FACILITIES AVAILABLE

7.1 Overview and Administration:

Stony Brook University Medical Center is the leading academic center on Long Island. There is a rich interactive atmosphere which includes researchers, clinicians, and a clinical trials group as well as support services for technical needs. Undergraduate, graduate, and medical students are available to participate in research. The hospital serves patients from both Nassau and Suffolk counties (population of 2.8 million). The neuroradiology department is in the same building with our center and offers expertise in MS and continuous consultative services. The neurology department provides the PI with 0.5 FTE secretarial support and clerical staff.

7.2 Research Facilities:

Dr. Krupp's research team consists of two neuropsychologists with statistical expertise and research associates skilled in neuropsychological testing. In the lab are eight computers with neuropsychological software programs, statistical (SAS, SPSS) and word processing packages. All data and study-related files are saved to the Clinical Research Center (CRC) server which is backed up daily and serviced by a 24 hour on-site contract with Dell. On-site CRC information technology support staff is also available. The CRC will provide has comfortable, well-lit rooms available for neuropsychological evaluations to be used in this study. The PI and her research team also have access to CRC nurses to assist with patient visits as needed.

7.3 Clinical:

Dr. Krupp is the Co-director of the Stony Brook University MS Comprehensive Care Center. The Center is affiliated with the local chapter of the National MS Society (NMSS). The MS center directors and staff (which include a nurse practitioner and a research nurse) have regular meetings to provide updates on clinical trials, recruitment, and clinical and administrative issues. Within the MS Center are databases with patient registries that also include information on cognition and can be used to facilitate recruitment for this study. Over 1000 patients are seen at the Center.

The PI meets frequently with the directors of the other MS Centers on Long Island. These physicians have been a source of referrals for prior investigator-initiated and industry-sponsored clinical trials. Dr. Patricia Coyle, Director of the MSCC, Dr. Myassar Zarif and Dr. Karen Blitz, two neurologists in private practice, have committed to assist with recruitment for this study. Each is confident they can refer approximately one patient per week from their practices.

LITERATURE CITED

1. Smith, A., Symbol Digits Modalities Test. 1982, Western Psychological Services: Los Angeles.
2. Smith, A., The symbol-digit modalities test: a neuropsychologic test of learning and other cerebral disorders, in Learning disorders, J. Helmuth, Editor. 1968, Special Child Publications: Seattle.
3. Benedict, R.H., Smerbeck, A., Parikh, R., Rodgers, J., Cadavid, D., and Erlanger, D., Reliability and equivalence of alternate forms for the Symbol Digit Modalities Test: implications for multiple sclerosis clinical trials. Mult Scler, 2012.
4. Batista, S., Zivadinov, R., Hoogs, M., Bergsland, N., Heininen-Brown, M., Dwyer, M.G., et al., Basal ganglia, thalamus and neocortical atrophy predicting slowed cognitive processing in multiple sclerosis. J Neurol, 2012. 259(1): p. 139-46.

5. Rao, S., and the Cognitive Function Study Group of the National Multiple Sclerosis Society, A manual for the brief repeatable battery of neuropsychological tests in multiple sclerosis. 1990, Section of Neuropsychology, Medical College of Wisconsin: Milwaukee, WI.
6. Gronwall, D.M., Paced auditory serial-addition task: a measure of recovery from concussion. Percept Mot Skills, 1977. 44(2): p. 367-73.
7. Vinogradov, S., Fisher, M., Holland, C., Shelly, W., Wolkowitz, O., and Mellon, S.H., Is serum brain-derived neurotrophic factor a biomarker for cognitive enhancement in schizophrenia? Biol Psychiatry, 2009. 66(6): p. 549-53.
8. Fisher, M., Holland, C., Merzenich, M.M., and Vinogradov, S., Using neuroplasticity-based auditory training to improve verbal memory in schizophrenia. Am J Psychiatry, 2009. 166(7): p. 805-11.
9. DeLuca, J., Barbieri-Berger, S., and Johnson, S.K., The nature of memory impairments in multiple sclerosis: acquisition versus retrieval. J Clin Exp Neuropsychol, 1994. 16(2): p. 183-9.
10. DeLuca, J., Gaudino, E.A., Diamond, B.J., Christodoulou, C., and Engel, R.A., Acquisition and storage deficits in multiple sclerosis. J Clin Exp Neuropsychol, 1998. 20(3): p. 376-90.
11. Tomassini, V., Johansen-Berg, H., Leonardi, L., Paixao, L., Jbabdi, S., Palace, J., et al., Preservation of motor skill learning in patients with multiple sclerosis. Mult Scler, 2011. 17(1): p. 103-15.
12. Benedict, R.H., Munschauer, F., Zarevics, P., Erlanger, D., Rowe, V., Feaster, T., et al., Effects of l-amphetamine sulfate on cognitive function in multiple sclerosis patients. J Neurol, 2008. 255(6): p. 848-52.
13. Grober, E., Ocepek-Welikson, K., Teresi, J., The Free and Cued Selective Reminding Test: evidence of psychometric adequacy. Psychology Science Quarterly, 2009. 51(3): p. 266-282.
14. Morrow, S.A., Kaushik, T., Zarevics, P., Erlanger, D., Bear, M.F., Munschauer, F.E., et al., The effects of L-amphetamine sulfate on cognition in MS patients: results of a randomized controlled trial. J Neurol, 2009. 256(7): p. 1095-102.
15. Sumowski, J.F., Chiaravalloti, N., Erlanger, D., Kaushik, T., Benedict, R.H., and DeLuca, J., L-amphetamine improves memory in MS patients with objective memory impairment. Mult Scler, 2011. 17(9): p. 1141-5.
16. Krupp, L.B., Christodoulou, C., Melville, P., Scherl, W.F., MacAllister, W.S., and Elkins, L.E., Donepezil improved memory in multiple sclerosis in a randomized clinical trial. Neurology, 2004. 63(9): p. 1579-85.
17. Benedict, R., Brief Visuospatial Memory Test – Revised: Professional Manual. 1997, Psychological Assessment Resources, Inc.: Odessa, FL.
18. Delis, D., Kaplan, E., Kramer, J., Delis-Kaplan Executive Function System. 2001, The Psychological Corporation, Harcourt Brace & Company: San Antonio.
19. Fisher, M., Holland, C., Subramaniam, K., and Vinogradov, S., Neuroplasticity-based cognitive training in schizophrenia: an interim report on the effects 6 months later. Schizophr Bull, 2010. 36(4): p. 869-79.
20. Barnes, D.E., Yaffe, K., Belfor, N., Jagust, W.J., DeCarli, C., Reed, B.R., et al., Computer-based cognitive training for mild cognitive impairment: results from a pilot randomized, controlled trial. Alzheimer Dis Assoc Disord, 2009. 23(3): p. 205-10.
21. Rosen, A., Sugiura, L., Belfor, N., Kramer, JH., Wagner, AD., Gabrieli, JD., An auditory perceptual approach to cognitive training in MCI: an fMRI study, in American Psychological Association Abstracts. 2008: San Francisco, CA.
22. Vance, D.E., Fazeli, P.L., Ross, L.A., Wadley, V.G., and Ball, K.K., Speed of Processing Training With Middle-Age and Older Adults With HIV: A Pilot Study. J Assoc Nurses AIDS Care, 2012.
23. U.S. Department of Defense Awards $2 Million to Brain Plasticity Inc. to Study Impact of Brain Training for Traumatic Brain Injuries, in PR Newswire United Business Media. June 21, 2011,

http://www.prnewswire.com/news-releases/us-department-of-defense-awards-2-million-to-brain-plasticity-inc-to-study-impact-of-brain-training-for-traumatic-brain-injuries-124277554.html.
24. Mahncke, H.W., Plasticity-Based Adaptive Cognitive Remediation for Schizophrenia. Brain Plasticity, Inc., 2010. NIH Project: 1RC3MH090833-01(http://projectreporter.nih.gov/project_info_details.cfm?aid=7926839&icde=13328376).
25. Lebowitz, M., Cantor, J., Gordon, W., Spielman, L., Ashman, T., Tsaousides, T., Spina, L., Examining the usability of a computerized cognitive training program in people with traumatic brain injury: a pilot study, in Archives of Physical Medicine and Rehabilitation. 2009: Denver, CO. p. e18-e19.
26. Krupp, L.B., Christodoulou, C., Melville, P., Scherl, W.F., Pai, L.Y., Muenz, L.R., et al., Multicenter randomized clinical trial of donepezil for memory impairment in multiple sclerosis. Neurology, 2011. 76(17): p. 1500-7.
27. Weinstein, A., Schwid, S.R., Schiffer, R.B., McDermott, M.P., Giang, D.W., and Goodman, A.D., Neuropsychologic status in multiple sclerosis after treatment with glatiramer. Arch Neurol, 1999. 56(3): p. 319-24.
28. Poser, C.M. and Brinar, V.V., The accuracy of prevalence rates of multiple sclerosis: a critical review. Neuroepidemiology, 2007. 29(3-4): p. 150-5.
29. Rao, S.M., Leo, G.J., Bernardin, L., and Unverzagt, F., Cognitive dysfunction in multiple sclerosis. I. Frequency, patterns, and prediction. Neurology, 1991. 41(5): p. 685-91.
30. Chiaravalloti, N.D. and DeLuca, J., Cognitive impairment in multiple sclerosis. Lancet Neurol, 2008. 7(12): p. 1139-51.
31. Rao, S.M., Leo, G.J., Ellington, L., Nauertz, T., Bernardin, L., and Unverzagt, F., Cognitive dysfunction in multiple sclerosis. II. Impact on employment and social functioning. Neurology, 1991. 41(5): p. 692-6.
32. Lyros, E., Messinis, L., Papageorgiou, S.G., and Papathanasopoulos, P., Cognitive dysfunction in multiple sclerosis: the effect of pharmacological interventions. Int Rev Psychiatry, 2010. 22(1): p. 35-42.
33. Benedict, R.H. and Zivadinov, R., Risk factors for and management of cognitive dysfunction in multiple sclerosis. Nat Rev Neurol, 2011. 7(6): p. 332-42.
34. Kappos, L., Freedman, M.S., Polman, C.H., Edan, G., Hartung, H.P., Miller, D.H., et al., Long-term effect of early treatment with interferon beta-1b after a first clinical event suggestive of multiple sclerosis: 5-year active treatment extension of the phase 3 BENEFIT trial. Lancet Neurol, 2009. 8(11): p. 987-97.
35. Fischer, J.S., Priore, R.L., Jacobs, L.D., Cookfair, D.L., Rudick, R.A., Herndon, R.M., et al., Neuropsychological effects of interferon beta-1a in relapsing multiple sclerosis. Multiple Sclerosis Collaborative Research Group. Ann Neurol, 2000. 48(6): p. 885-92.
36. Pliskin, N.H., Hamer, D.P., Goldstein, D.S., Towle, V.L., Reder, A.T., Noronha, A., et al., Improved delayed visual reproduction test performance in multiple sclerosis patients receiving interferon beta-1b. Neurology, 1996. 47(6): p. 1463-8.
37. Qu, Z.X., Pliskin, N., Jensen, M.W., White, D., and Arnason, B.G., Etretinate augments interferon beta-1b effects on suppressor cells in multiple sclerosis. Arch Neurol, 2001. 58(1): p. 87-90.
38. Cicerone, K.D., Langenbahn, D.M., Braden, C., Malec, J.F., Kalmar, K., Fraas, M., et al., Evidence-based cognitive rehabilitation: updated review of the literature from 2003 through 2008. Arch Phys Med Rehabil, 2011. 92(4): p. 519-30.
39. Wykes, T., Huddy, V., Cellard, C., McGurk, S.R., and Czobor, P., A meta-analysis of cognitive remediation for schizophrenia: methodology and effect sizes. Am J Psychiatry, 2011. 168(5): p. 472-85.

40. O'Brien, A.R., Chiaravalloti, N., Goverover, Y., and Deluca, J., Evidenced-based cognitive rehabilitation for persons with multiple sclerosis: a review of the literature. Arch Phys Med Rehabil, 2008. 89(4): p. 761-9.
41. Rosti-Otajarvi, E.M. and Hamalainen, P.I., Neuropsychological rehabilitation for multiple sclerosis. Cochrane Database Syst Rev, 2011. 11: p. CD009131.
42. Mattioli, F., Stampatori, C., Zanotti, D., Parrinello, G., and Capra, R., Efficacy and specificity of intensive cognitive rehabilitation of attention and executive functions in multiple sclerosis. J Neurol Sci, 2010. 288(1-2): p. 101-5.
43. Langdon, D., Cognitive rehabilitation in multiple sclerosis, in Multiple Sclerosis: Recovery of Function and Neurorehabilitation, J. Kesselring, Comi, G., Thompson, A., Editor. 2010, Cambridge University Press.
44. Shatil, E., Metzer, A., Horvitz, O., and Miller, A., Home-based personalized cognitive training in MS patients: a study of adherence and cognitive performance. NeuroRehabilitation, 2010. 26(2): p. 143-53.
45. Sastre-Garriga, J., Alonso, J., Renom, M., Arevalo, M.J., Gonzalez, I., Galan, I., et al., A functional magnetic resonance proof of concept pilot trial of cognitive rehabilitation in multiple sclerosis. Mult Scler, 2011. 17(4): p. 457-67.
46. Plohmann, A.M., Kappos, L., Ammann, W., Thordai, A., Wittwer, A., Huber, S., et al., Computer assisted retraining of attentional impairments in patients with multiple sclerosis. J Neurol Neurosurg Psychiatry, 1998. 64(4): p. 455-62.
47. Chiaravalloti, N.D., DeLuca, J., Moore, N.B., and Ricker, J.H., Treating learning impairments improves memory performance in multiple sclerosis: a randomized clinical trial. Mult Scler, 2005. 11(1): p. 58-68.
48. Hildebrandt, H., Lanz, M., Hahn, H.K., Hoffmann, E., Schwarze, B., Schwendemann, G., et al., Cognitive training in MS: effects and relation to brain atrophy. Restor Neurol Neurosci, 2007. 25(1): p. 33-43.
49. Messinis, L., Kosmidis, M.H., Lyros, E., and Papathanasopoulos, P., Assessment and rehabilitation of cognitive impairment in multiple sclerosis. Int Rev Psychiatry, 2010. 22(1): p. 22-34.
50. Tesar, N., Bandion, K., and Baumhackl, U., Efficacy of a neuropsychological training programme for patients with multiple sclerosis -- a randomised controlled trial. Wien Klin Wochenschr, 2005. 117(21-22): p. 747-54.
51. Buonomano, D.V. and Merzenich, M.M., Cortical plasticity: from synapses to maps. Annu Rev Neurosci, 1998. 21: p. 149-86.
52. Kessels, R.P. and de Haan, E.H., Implicit learning in memory rehabilitation: a meta-analysis on errorless learning and vanishing cues methods. J Clin Exp Neuropsychol, 2003. 25(6): p. 805-14.
53. Squire, L.R. and Zola, S.M., Structure and function of declarative and nondeclarative memory systems. Proc Natl Acad Sci U S A, 1996. 93(24): p. 13515-22.
54. Schmitter-Edgecombe, M., Implications of basic science research for brain injury rehabilitation: a focus on intact learning mechanisms. J Head Trauma Rehabil, 2006. 21(2): p. 131-41.
55. Merzenich, M., Jenkins, WM., Cortical plasticity, learning and learning dysfunction, in Maturational Windows and Adult Cortical Plasticity. 1995, Westview Press. p. 247-272.
56. Merzenich, M., Tallal, P., Peterson B., Miller S., Jenkins, WM., Some neurological principles relevant to the origins of - and the cortical plasticity-based remediation of - developmental language impairments, in Neuronal plasticity: building a bridge from the laboratory to the clinic. 1999, Springer. p. 169-187.
57. Merzenich, M., Cortical plasticity contributing to child development, in Mechanisms of cognitive development: Behavioral and neural perspectives. 2001, Psychology Press. p. 67-95.

58. Recanzone, G.H., Merzenich, M.M., and Schreiner, C.E., Changes in the distributed temporal response properties of SI cortical neurons reflect improvements in performance on a temporally based tactile discrimination task. J Neurophysiol, 1992. 67(5): p. 1071-91.
59. Recanzone, G.H., Jenkins, W.M., Hradek, G.T., and Merzenich, M.M., Progressive improvement in discriminative abilities in adult owl monkeys performing a tactile frequency discrimination task. J Neurophysiol, 1992. 67(5): p. 1015-30.
60. Tallal, P., Miller, S.L., Bedi, G., Byma, G., Wang, X., Nagarajan, S.S., et al., Language comprehension in language-learning impaired children improved with acoustically modified speech. Science, 1996. 271(5245): p. 81-4.
61. Merzenich, M., Wright, BA., Jenkins, W., Xerri, C., Yle, N., Miller, S., Tallal, P., Cortical plasticity underlying perceptual, motor, and cognitive skill development: implications for neurorehabilitation, in Brain development and cognition: a reader. 2002, Wiley-Blackwell. p. 293-304.
62. Cifu, D.X., Cohen, S.I., Lew, H.L., Jaffee, M., and Sigford, B., The history and evolution of traumatic brain injury rehabilitation in military service members and veterans. Am J Phys Med Rehabil, 2010. 89(8): p. 688-94.
63. Taber, K.H., Warden, D.L., and Hurley, R.A., Blast-related traumatic brain injury: what is known? J Neuropsychiatry Clin Neurosci, 2006. 18(2): p. 141-5.
64. Cicerone, K.D., Dahlberg, C., Kalmar, K., Langenbahn, D.M., Malec, J.F., Bergquist, T.F., et al., Evidence-based cognitive rehabilitation: recommendations for clinical practice. Arch Phys Med Rehabil, 2000. 81(12): p. 1596-615.
65. Cicerone, K.D., Dahlberg, C., Malec, J.F., Langenbahn, D.M., Felicetti, T., Kneipp, S., et al., Evidence-based cognitive rehabilitation: updated review of the literature from 1998 through 2002. Arch Phys Med Rehabil, 2005. 86(8): p. 1681-92.
66. Sohlberg, M., Avery, JA., Kennedy, M., Ylvisaker, M., Coelho, C., Turkstra, L., Yorkston, K., Practice guidelines for direct attention training. Journal of Medical Speech-Language Pathology, 2003. 11(3): p. xix-xxxix.
67. Hoge, C.W., McGurk, D., Thomas, J.L., Cox, A.L., Engel, C.C., and Castro, C.A., Mild traumatic brain injury in U.S. Soldiers returning from Iraq. N Engl J Med, 2008. 358(5): p. 453-63.
68. Terrio, H., Brenner, L.A., Ivins, B.J., Cho, J.M., Helmick, K., Schwab, K., et al., Traumatic brain injury screening: preliminary findings in a US Army Brigade Combat Team. J Head Trauma Rehabil, 2009. 24(1): p. 14-23.
69. Kaschel, R., Della Sala, S., Cantagallo, A., Fahlböck, A., Laaksonen, R., Kazen, M., Imagery mnemonics for the rehabilitation of memory: A randomised group controlled trial. Neuropsychological Rehabilitation, 2002. 12(2): p. 127-153.
70. Merzenich, M., Jenkins, WM., Cortical representation of learned behaviors, in Memory Concepts: Basic and Clinical Aspects: Proceedings of the 7th Novo Nordisk Foundation Symposium. 1993, Excerpta Medica: Copenhagen, Denmark. p. 437.
71. Merzenich, M., DeCharms, RC., Neural representations, experience and change, in Mind-Brain Continuum. 1996, MIT Press: Cambridge, MS. p. 61-81.
72. Scalf, P.E., Colcombe, S.J., McCarley, J.S., Erickson, K.I., Alvarado, M., Kim, J.S., et al., The neural correlates of an expanded functional field of view. J Gerontol B Psychol Sci Soc Sci, 2007. 62 Spec No 1: p. 32-44.
73. Berry, A.S., Zanto, T.P., Clapp, W.C., Hardy, J.L., Delahunt, P.B., Mahncke, H.W., et al., The influence of perceptual training on working memory in older adults. PLoS One, 2010. 5(7): p. e11537.

74. Vinogradov, S., Fisher, M., Warm, H., Holland, C., Kirshner, M.A., and Pollock, B.G., The cognitive cost of anticholinergic burden: decreased response to cognitive training in schizophrenia. Am J Psychiatry, 2009. 166(9): p. 1055-62.
75. Smith, G.E., Housen, P., Yaffe, K., Ruff, R., Kennison, R.F., Mahncke, H.W., et al., A cognitive training program based on principles of brain plasticity: results from the Improvement in Memory with Plasticity-based Adaptive Cognitive Training (IMPACT) study. J Am Geriatr Soc, 2009. 57(4): p. 594-603.
76. Edwards, J.D., Wadley, V.G., Myers, R.S., Roenker, D.L., Cissell, G.M., and Ball, K.K., Transfer of a speed of processing intervention to near and far cognitive functions. Gerontology, 2002. 48(5): p. 329-40.
77. Edwards, J.D., Wadley, V.G., Vance, D.E., Wood, K., Roenker, D.L., and Ball, K.K., The impact of speed of processing training on cognitive and everyday performance. Aging Ment Health, 2005. 9(3): p. 262-71.
78. Roenker, D.L., Cissell, G.M., Ball, K.K., Wadley, V.G., and Edwards, J.D., Speed-of-processing and driving simulator training result in improved driving performance. Hum Factors, 2003. 45(2): p. 218-33.
79. Keefe, R.S., Fox, K.H., Harvey, P.D., Cucchiaro, J., Siu, C., and Loebel, A., Characteristics of the MATRICS Consensus Cognitive Battery in a 29-site antipsychotic schizophrenia clinical trial. Schizophr Res, 2011. 125(2-3): p. 161-8.
80. Dickinson, D., Tenhula, W., Morris, S., Brown, C., Peer, J., Spencer, K., et al., A randomized, controlled trial of computer-assisted cognitive remediation for schizophrenia. Am J Psychiatry, 2010. 167(2): p. 170-80.
81. Popov, T., Jordanov, T., Rockstroh, B., Elbert, T., Merzenich, M.M., and Miller, G.A., Specific cognitive training normalizes auditory sensory gating in schizophrenia: a randomized trial. Biol Psychiatry, 2011. 69(5): p. 465-71.
82. Delis, D., Kramer, JH., Kaplan, E., Ober, BA., California Verbal Learning Test (2nd ed.). 2000, Psychological Corporation: San Antonio, TX.
83. Polman, C.H., Reingold, S.C., Banwell, B., Clanet, M., Cohen, J.A., Filippi, M., et al., Diagnostic criteria for multiple sclerosis: 2010 revisions to the McDonald criteria. Ann Neurol, 2011. 69(2): p. 292-302.
84. Stone, M., Jastak, S., Wilkinson, G., Wide Range Achievement Test - 3. Rasch Measurement Transactions, 1995. 8(4): p. 403.
85. Rosti, E., Hamalainen, P., Koivisto, K., and Hokkanen, L., PASAT in detecting cognitive impairment in relapsing-remitting MS. Appl Neuropsychol, 2007. 14(2): p. 101-12.
86. Mahncke, H.W., Connor, B.B., Appelman, J., Ahsanuddin, O.N., Hardy, J.L., Wood, R.A., et al., Memory enhancement in healthy older adults using a brain plasticity-based training program: a randomized, controlled study. Proc Natl Acad Sci U S A, 2006. 103(33): p. 12523-8.
87. Hoyle Puzzle and Board Games, http://www.hoylegaming.com. 2011, Navarre Corporation Company.
88. Sepama Software, www.track4win.com. 2000.
89. Wechsler, D., Coalson, DL., Raiford, SE., WAIS-IV technical and interpretive manual. 2008, Pearson: San Antonio, TX.
90. Buschke, H. and Fuld, P.A., Evaluating storage, retention, and retrieval in disordered memory and learning. Neurology, 1974. 24(11): p. 1019-25.
91. Scherl, W.F., Krupp, L.B., Christodoulou, C., Morgan, T.M., Hyman, L., Chandler, B., et al., Normative data for the selective reminding test: a random digit dialing sample. Psychol Rep, 2004. 95(2): p. 593-603.

92. Mathiowetz, V., Weber, K., Kashman, B., Volland, G., Adult norms for the nine hole pef test of finger dexterity. Am J Occup Ther, 1985. 5: p. 24-38.
93. Cutter, G.R., Baier, M.L., Rudick, R.A., Cookfair, D.L., Fischer, J.S., Petkau, J., et al., Development of a multiple sclerosis functional composite as a clinical trial outcome measure. Brain, 1999. 122 (Pt 5): p. 871-82.
94. Owsley, C., McGwin, G., Jr., Sloane, M.E., Stalvey, B.T., and Wells, J., Timed instrumental activities of daily living tasks: relationship to visual function in older adults. Optom Vis Sci, 2001. 78(5): p. 350-9.
95. Owsley, C., Sloane, M., McGwin, G., Jr., and Ball, K., Timed instrumental activities of daily living tasks: relationship to cognitive function and everyday performance assessments in older adults. Gerontology, 2002. 48(4): p. 254-65.
96. Goverover, Y., Genova, H.M., Hillary, F.G., and DeLuca, J., The relationship between neuropsychological measures and the Timed Instrumental Activities of Daily Living task in multiple sclerosis. Mult Scler, 2007. 13(5): p. 636-44.
97. Wadley, V.G., Okonkwo, O., Crowe, M., and Ross-Meadows, L.A., Mild cognitive impairment and everyday function: evidence of reduced speed in performing instrumental activities of daily living. Am J Geriatr Psychiatry, 2008. 16(5): p. 416-24.
98. Goverover, Y., O'Brien, A.R., Moore, N.B., and DeLuca, J., Actual reality: a new approach to functional assessment in persons with multiple sclerosis. Arch Phys Med Rehabil, 2010. 91(2): p. 252-60.
99. Ventura, J., Reise, S.P., Keefe, R.S., Baade, L.E., Gold, J.M., Green, M.F., et al., The Cognitive Assessment Interview (CAI): development and validation of an empirically derived, brief interview-based measure of cognition. Schizophr Res, 2010. 121(1-3): p. 24-31.
100. O'Brien, A., Gaudino-Goering, E., Shawaryn, M., Komaroff, E., Moore, N.B., and DeLuca, J., Relationship of the Multiple Sclerosis Neuropsychological Questionnaire (MSNQ) to functional, emotional, and neuropsychological outcomes. Arch Clin Neuropsychol, 2007. 22(8): p. 933-48.
101. Ruff, R., Hibbard, K., Ruff Neurobehavioral Inventory: Professional Manual. 2003, Psychological Assessment Resources, Inc.: Odessa, FL.
102. Yuan, Y., Multiple Imputation for Missing data: Concepts and New Development, paper. April 9-12, 2000, SUGI25 (SAS User's Group International): Indianapolis, Indiana.
103. Jennison, C., Turnbull, BW., Section 2.5, in Group sequential methods with applications to clinical trials. 2000, Chapman & Hall: Boca Raton.
104. Jennison, C., Turnbull, BW., Section 10.2, in Group sequential methods with applications to clinical trials. 2000, Chapman & Hall: Boca Raton.
