# Supplementary figures and images for: Cognitive function in multiple sclerosis improves with telerehabilitation: Results from a randomized controlled trial
Source: PLoS One. 2017 May 11;12(5):e0177177. doi: 10.1371/journal.pone.0177177 (PMC5426671; doi:10.1371/journal.pone.0177177)

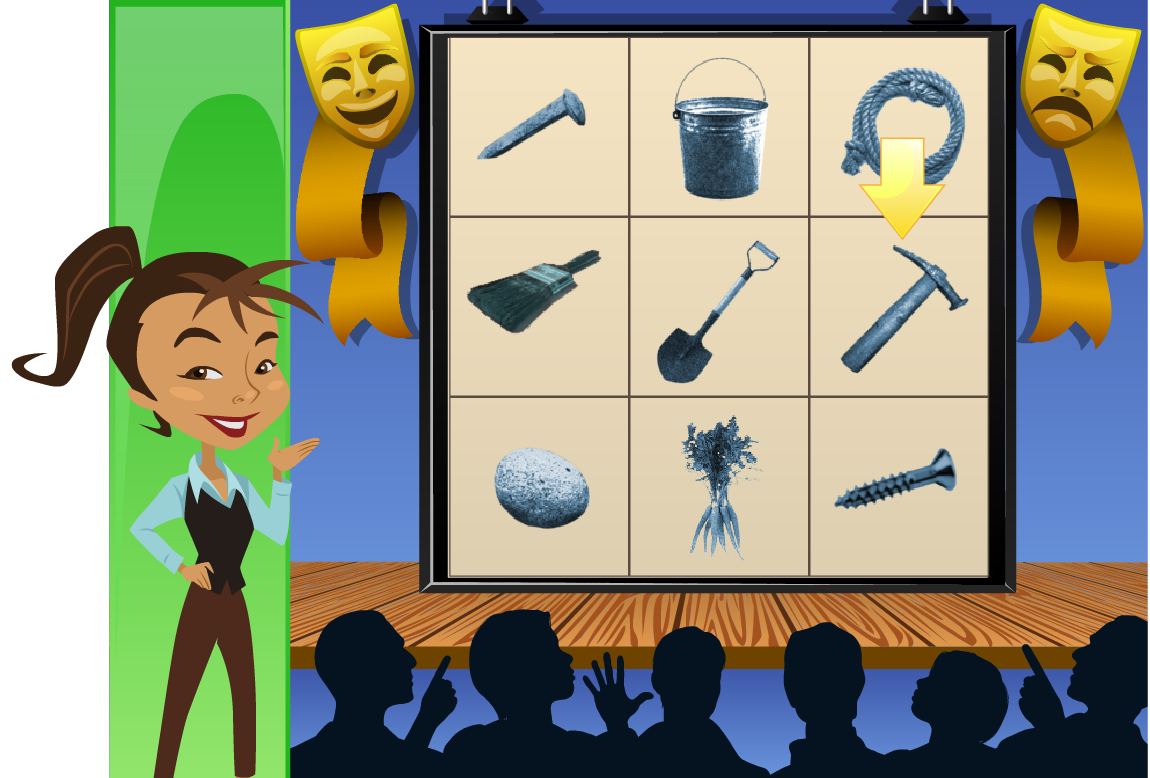

Supplement: S1 Screenshot — (TIFF) [file pone.0177177.s004.tiff]

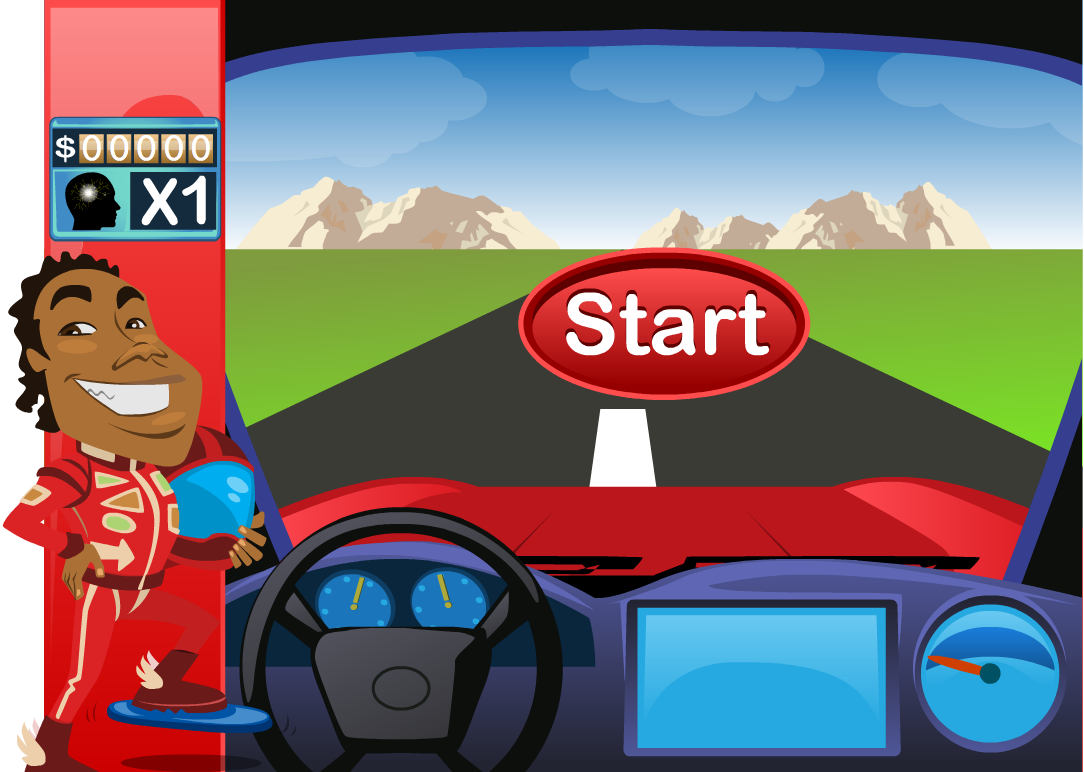

Supplement: S2 Screenshot — (TIFF) [file pone.0177177.s005.tiff]

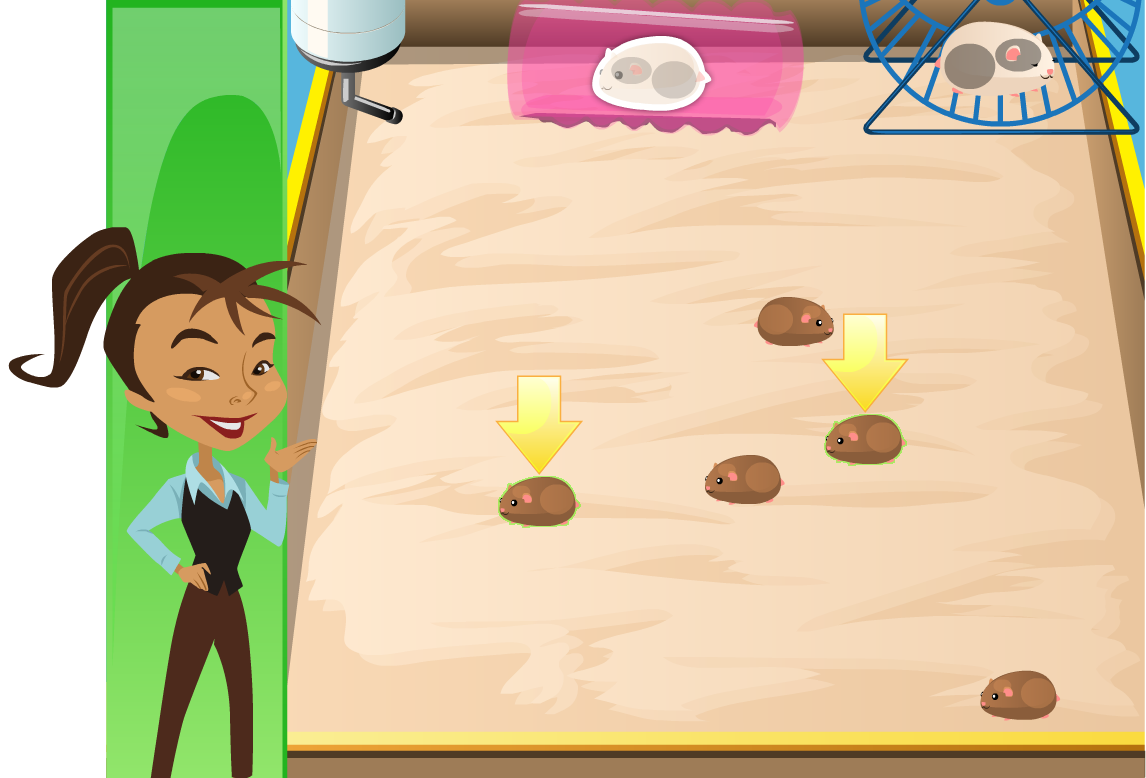

Supplement: S3 Screenshot — (TIFF) [file pone.0177177.s006.tiff]
